# The Complex Spatio-Temporal Regulation of the *Drosophila* Myoblast Attractant Gene *duf/kirre*

**DOI:** 10.1371/journal.pone.0006960

**Published:** 2009-09-09

**Authors:** K. G. Guruharsha, Mar Ruiz-Gomez, H. A. Ranganath, Rahul Siddharthan, K. VijayRaghavan

**Affiliations:** 1 National Centre for Biological Sciences, Tata Institute of Fundamental Research, Bangalore, India; 2 Department of Studies in Zoology, University of Mysore, Manasagangothri, Mysore, India; 3 Centro de Biologia Molecular Severo Ochoa, CSIC and UAM, Cantoblanco, Madrid, Spain; 4 Institute of Mathematical Sciences, CIT Campus, Taramani, Chennai, India; Institute of Genetics and Molecular and Cellular Biology, France

## Abstract

A key early player in the regulation of myoblast fusion is the gene *dumbfounded* (*duf,* also known as *kirre*). Duf must be expressed, and function, in founder cells (FCs). A fixed number of FCs are chosen from a pool of equivalent myoblasts and serve to attract fusion-competent myoblasts (FCMs) to fuse with them to form a multinucleate muscle-fibre. The spatial and temporal regulation of *duf* expression and function are important and play a deciding role in choice of fibre number, location and perhaps size. We have used a combination of bioinformatics and functional enhancer deletion approaches to understand the regulation of *duf*. By transgenic enhancer-reporter deletion analysis of the *duf* regulatory region, we found that several distinct enhancer modules regulate *duf* expression in specific muscle founders of the embryo and the adult. In addition to existing bioinformatics tools, we used a new program for analysis of regulatory sequence, PhyloGibbs-MP, whose development was largely motivated by the requirements of this work. The results complement our deletion analysis by identifying transcription factors whose predicted binding regions match with our deletion constructs. Experimental evidence for the relevance of some of these TF binding sites comes from available ChIP-on-chip from the literature, and from our analysis of localization of myogenic transcription factors with *duf* enhancer reporter gene expression. Our results demonstrate the complex regulation in each founder cell of a gene that is expressed in all founder cells. They provide evidence for transcriptional control—both activation and repression—as an important player in the regulation of myoblast fusion. The set of enhancer constructs generated will be valuable in identifying novel *trans-*acting factor-binding sites and chromatin regulation during myoblast fusion in *Drosophila*. Our results and the bioinformatics tools developed provide a basis for the study of the transcriptional regulation of other complex genes.

## Introduction

Multinucleate muscle fibres form by the regulated fusion of myoblasts. Muscles of different shapes and sizes are made as a result of coordinated myoblast fusion and morphogenesis. This process is perhaps best studied in the embryonic muscles of the fruitfly *Drosophila melanogaster* (reviewed in [1]). Within myogenic domains, myoblasts are separated into founder cells (FCs) and fusion-competent myoblasts (FCMs) by *Notch (N)* mediated lateral inhibition and other signaling pathways [2], [3]. FCs seed the formation of muscles by attracting FCMs to fuse and form multi-nucleate fibres in a defined pattern. The mutual recognition of FCs and FCMs is mediated by a group of transmembrane proteins belonging to the immunoglobulin superfamily. One of these, Dumbfounded (Duf, also called Kirre), marks the surface of FCs [4] and another, Sticks and stones (Sns) the complementary subset of FCMs [5]. When examined by mRNA *in situ* hybridization or reporter-gene expression, *sns* and *duf* are expressed transiently in FCs and FCMs respectively and are turned off soon after the fusion process is complete [4], [5]. This suggests that *duf* and *sns* are subject to strong transcriptional regulation.

In the *Drosophila* adult and invertebrates, muscles consist of many myotubes bundled together to form a contractile element. *Drosophila* adult muscle precursor cells segregate as sister cells from embryonic founders and give rise to all adult muscles in the pupa [6], [7]. These adult myoblasts maintain *twi* expression, proliferate during larval life and remain associated with imaginal discs in the thorax and neurons in the abdomen [8]. Specific myoblast groups are chosen to give rise to different muscles under the influence of signaling molecules and transcription factors. Apterous (Ap) and Cut (Ct) are important for direct flight muscles development [9]; Vestigial (Vg) and Cut (Ct), regulated by Wingless (Wg), are responsible for indirect flight muscle development [10]. Unlike in the embryo, Notch-mediated lateral inhibition is not involved in founder cell specification during adult thoracic myogenesis [11]. However, as in the embryo [12], [13], components of the Fibroblast growth factor (FGF) pathway mediates founder cell choice [14]. This results in a precise pattern of founder cells for each multi-fibre array of adult abdominal muscles. Expression of myoblast fusion genes is transient and tightly regulated in adult founder analogs too [14].

The size of the muscle fibre is probably dependent upon the number of fusion events [15]. The duration and level of Duf/Kirre on the FC membrane along with other fusion proteins, especially Rolling pebbles 7 (Rols7; also known as Antisocial), [16], [17] appears to regulate this mechanism. Duf has been shown to be a rate-limiting factor in myoblast fusion during embryonic myogenesis [18]. Characterization of enhancer sequences of *duf* is therefore important to understand the transcriptional machinery that recognizes a FC. This will also allow us to understand the role of different factors responsible for transcriptional control of *duf* in different FCs and thereby development of muscle pattern.

Bioinformatics tools can predict possible transcription factor (TF) binding sites, either by comparing with previously identified sites for known TFs, or *ab initio* by looking for short inexact repeated patterns or “motifs”. Enhancers and *cis*-regulatory modules can be predicted by clustering predicted binding sites, an approach taken by programs such as Stubb [19], [20], eCis-Analyst [21], [22] and Cluster-Buster [23]. Recently, with the availability of sequence information from related species including twelve *Drosophila* genomes [24], new approaches have been developed to make use of orthologous sequence from related species to specific region of interest. A simple approach is “phylogenetic footprinting” [25], [26], [27], which confines searches to sequences that are highly conserved across species, using the assumption that such regions are more likely to be functional. However, it is also known that gene regulation evolves significantly even among closely related species, and binding sites that are known to be functional in one species disappear or are replaced by new sites in other species (for example see [28]). Therefore, some newer programs, including PhyloGibbs [29] (a motif-finder) and Stubb [19], [20] (a module-prediction program), both of which we have used in this study, analyse both conserved and non-conserved sequences but modify scoring to take into account phylogenetic relationship between species. However, these approaches cannot by themselves give a sense of the temporal or spatial aspects of gene regulation. To be effective, they must be combined with prior experimental information about the transcriptional regulation and spatio- temporal expression pattern of the genes of interest. For example, a recent [30] study made use of known spatio- temporal concentrations of transcription factors to predict the expression levels of cis-regulatory modules in the segmentation network. Unfortunately such prior data is a luxury often unavailable.

We have used a combination of bioinformatics and functional enhancer deletion approaches to understand the regulation of *duf*. In addition to existing bioinformatics tools, we used a new program for analysis of regulatory sequence, PhyloGibbs-MP, described in a recent paper [31], whose development was largely motivated by the requirements of this work. Studies with Stubb [19], [20] using published consensus sequences for mesoderm relevant factors found evidence of a modular structure of enhancers both upstream of the gene and in its intron. Deletion analysis of the *duf* regulatory region using reporter constructs reveals specific aspects of *duf* regulation during *Drosophila* myogenesis. We find that several distinct enhancer modules regulate *duf* expression in specific muscle founders of the embryo and the adult. While embryonic enhancers are proximal, adult-specific enhancers are located more distal to the *duf* start site. These results merited a further, detailed study of the 10 kb region upstream of *duf*. We made a list of 44 position weight matrices for transcription factors relevant to mesoderm development, of which 38 were constructed using either the FlyReg database for DNAse I footprints [32] or recent data from bacteria-one-hybrid systems [33] and 6 were taken from the literature. We rejected 13 as not being specific enough or not showing significant predictions in preliminary runs. We used the remaining 31 matrices with two programs, Stubb [19], [20] and PhyloGibbs-MP [31], to predict *cis*-regulatory modules as well as binding sites for individual transcription factors. The results complement our deletion analysis by identifying transcription factors whose predicted binding regions match with our deletion constructs. Most predicted sites are conserved in other *Drosophila* species, suggesting functional importance. Experimental evidence for the relevance of some of these TF binding sites comes from both specific and global ChIP-on-chip analysis from the literature, using key mesodermal regulators [34], [35], [36]. Though bioinformatic predictive tools and ChIP-on-chip approaches are unable, by themselves, to predict the full spatio-temporal behaviour of gene regulation, we demonstrate their utility when combined with our experimental information. Given the many conserved aspects of myogenic regulation between flies and vertebrates, recently underscored by the demonstration of the role of kirre in zebrafish [37], our results are likely to be of broad value.

## Results

### Identification of *duf* Enhancer Region

Preliminary studies of the 40 kb region upstream of *duf* and of the first intron (29 kb) were made using Matinspector Professional® [38]. MatInspector is a tool for transcription factor binding site analysis by Genomatix which utilizes its own transcription factor knowledge base (MatBase) to locate transcription factor binding sites in sequences of any length. Additional binding sites information for nuclear effectors of important signaling pathways and mesoderm specific factors from published work that were not available in Matinspector Professional® were integrated into our search for *cis-*regulatory elements regulating *duf* expression in FCs. The consensus sequences (and source) for these factors are described in Materials and Methods.

**Figure 1:**
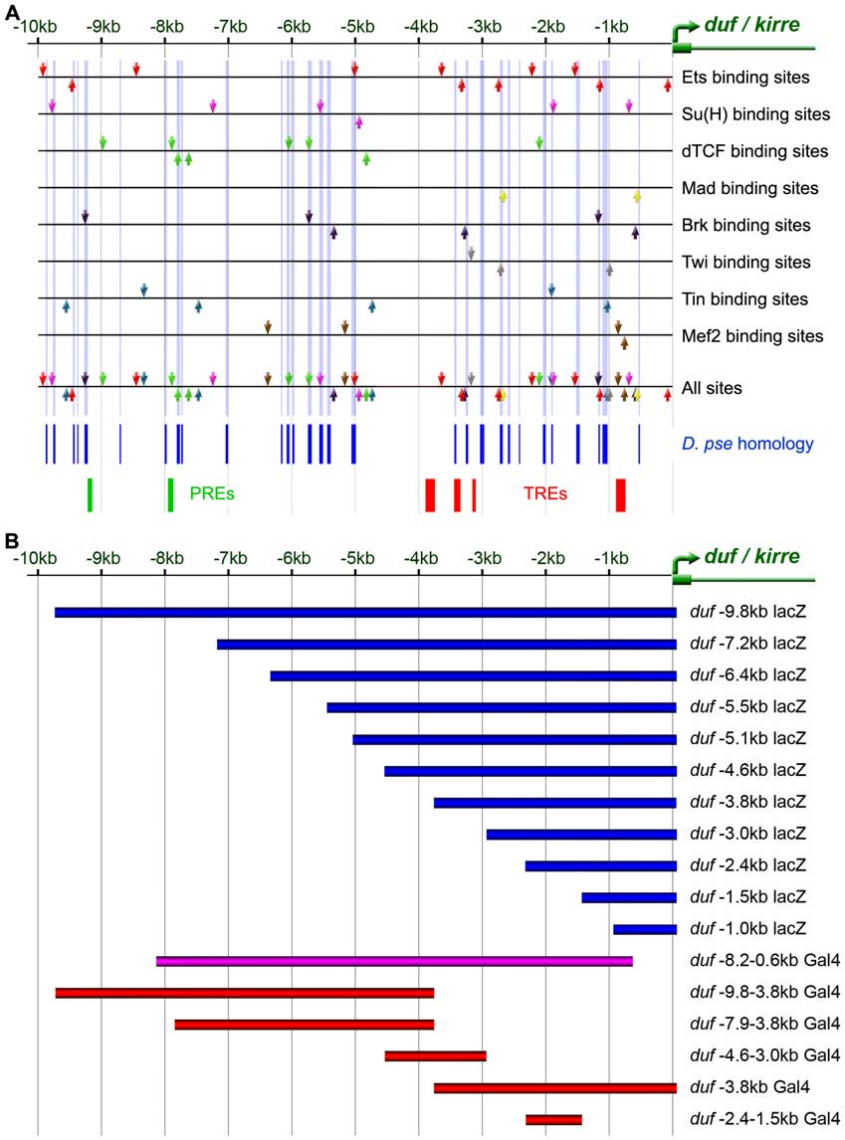
Consensus binding sites in *duf* enhancer sequences and deletion analysis of *duf* genomic region. A. Occurrence of binding sites for nuclear effectors of signaling pathways and mesodermal factors in the 10 kb sequence 5′ to *duf* is diagrammatically shown. Published consensus binding sequences for Ets, Su(H), Ci, dTCF, Mad, Brk, Twi, Tin, Mef2 are shown. Downward pointing arrow indicates binding on + strand and upward pointing arrow indicates binding on –strand. Sequences that are well conserved in *D. pseudoobscura* are shown as blue vertical bars. Several putative binding sites for GAGA factor encoded by the Trithorax-like gene (Trl) characteristic of TREs (Trithorax Response Elements) are present within 3.8 kb from the *duf* start site (red verticle bars) but no sites further upstream in the 10 kb region. Similarly, putative binding sites for PHO (pleiohomeotic) and PHO-like, polycomp group proteins (PcG) that bind to PREs (Polycomb group Response Elements) are found between −8.0 kb to −9.3 kb region (green vertical bars). B. Schematic of constructs generated to characterize the regulatory potential of putative *duf* enhancer sequences during *Drosophila* embryonic and adult myogenesis. Putative enhnacer fragments with deletions from both distal (blue bars) and proximal ends (red bars) of the *duf* 5′ region were PCR amplified and cloned as EcoRI - BamHI fragments into pCaSpeR AUG βGal, or pPTGal. Transgenic flies were generated from these constructs producing either lacZ (blue bars) or Gal4 lines (red bars). *duf* −8.2−0.6 was cloned into ZGLpWW vector (magenta bar).

Analysis of this sequence revealed presence of many strong binding sites for nuclear effectors of different intercellular signaling pathways. Some clustering of binding sites was seen in the 10 kb region immediately upstream of *duf*. The arrangement of these putative binding sites in *duf* upstream region is shown in Figure 1A for select factors. Distinct PREs (Polycomb group Response Elements) and TREs (Trithorax Response Elements) are also found in this region (Figure 1A). The list of factors and their putative binding sites are listed in Supplementary Datasheet S1.

The putative binding sites were compared using sequence similarity between *Drosophila melanogaster* and *Drosophila pseudoobscura* genomes. A significant number of the putative binding sites for signaling pathway effectors and transcription factors with mesodermal role and early patterning genes map within or in the vicinity of conserved sequence stretches (vertical blue bars Figure 1A). The results are tabulated for all TF binding sites in Supplementary Datasheet S2 and summarized for key signaling pathway effectors and mesodermal factors in Figure 1A.

To characterize the regulatory potential of putative *duf* enhancer sequences, chosen genomic sequences were amplified by the polymerase chain reaction (PCR) from the *duf* 5′ region. Fragments representing progressive deletions from both distal and proximal ends were amplified and cloned as *EcoRI - BamHI* fragments into pCaSpeR AUG βGal [39], or pPTGal [40]. Transgenic flies were generated from these constructs producing either lacZ (blue bars in Figure 1B) or Gal4 lines (red bars in Figure 1B) as described in Materials and Methods. The expression patterns of these *duf* enhancer constructs were analyzed for reporter expression during embryonic and adult myogenesis. Additional *duf* enhancer-deletion reporter constructs for further analysis (results not discussed) are in Supplementary data Figure S1.

### Modular Enhancers Regulate *duf* Expression during Embryonic Myogenesis

**Figure 2:**
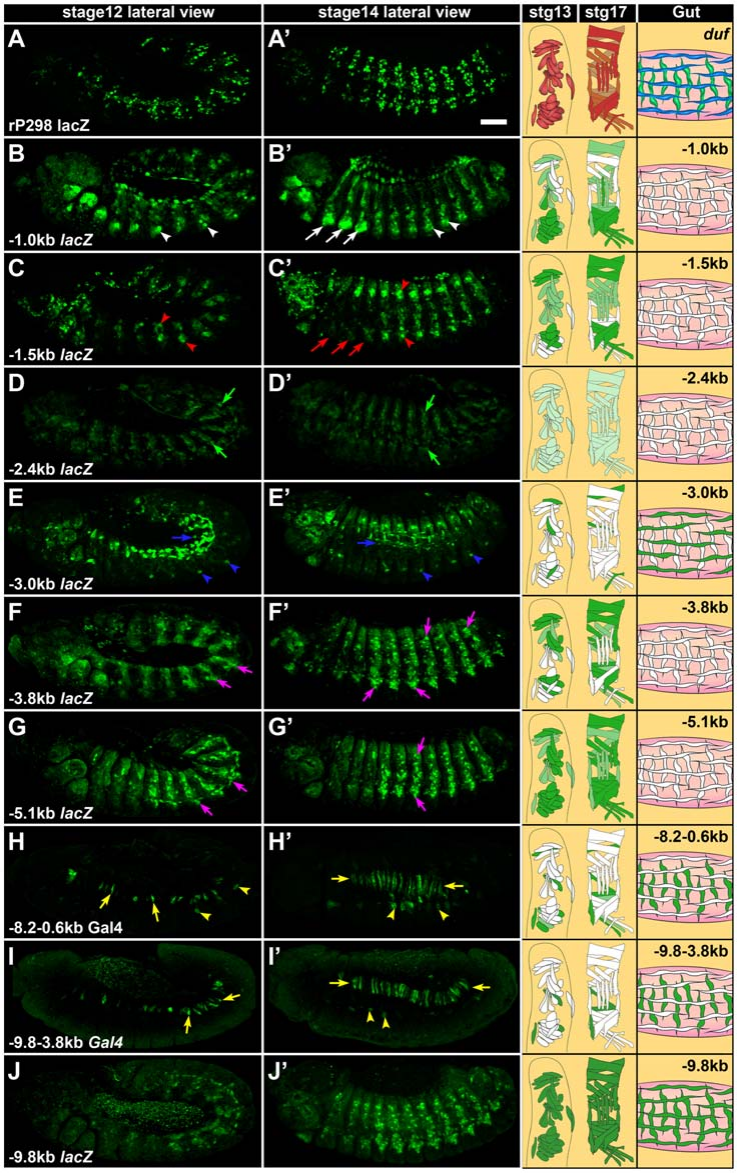
Modular enhancers regulate *duf* expression during embryonic myogenesis. Confocal projections of different *duf* enhancer reporter expression assayed using anti β gal staining in *Drosophila* embryos. Level of reporter expression in muscles is represented by intensity of green colour in the cartoon to the right. Wildtype *duf* expression is visualized using *rP298 lacZ* (A and A′). *duf −1.0 kb lacZ* is expressed in few somatic FCs (arrowheads in B and B′) and ectopically in ventral thoracic region (arrows in B and B′), which is repressed in *duf −1.5 kb lacZ* (arrows in C and C′). *duf −1.5 kb lacZ* is expressed in ventral and dorsal abdominal somatic FCs (arrowheads in C and C′) and weakly in lateral somatic FCs. *duf* −*2.4 kb lacZ* is expressed very weakly in somatic FCs (arrows in D and D′). *duf −3.0 kb lacZ* is specifically expressed in longitudinal visceral FCs (arrow in E) that align longitudinally around the gut following fusion with FCMs (arrow in E′). It is also expressed in 3 somatic FCs (arrowheads in E and E′) but not in circular visceral muscles. *duf −3.8 kb lacZ* and *duf −5.1 kb lacZ* are expressed in a large subset of somatic FCs (arrows in F–G′) but not in visceral mesoderm. *duf −8.2 −0.6 kb Gal4* and *duf −9.8−3.8 kb Gal4* marks the circular visceral muscle FCs (arrows in H–I′) and few ventral somatic muscle FCs (arrowheads in H–I′). *duf −9.8 kb lacZ* recapitulates the complete wildtype *duf* expression and marks all the embryonic muscle FCs. All embryos are lateral view with anterior to the left and dorsal to the top. Scale bar = 50 microns.

The dynamics of wildtype *duf* expression during embryonic muscle development has been characterized using mRNA in-situ hybridizations [4] and by the use of *rP298 lacZ*, [41] a P- nuclear lacZ insertion into the *duf* locus, which reproduces *duf* -like reporter- expression in all founder cells during embryonic [4] and adult myogenesis [11]. The expression pattern of different *duf* upstream reporter constructs were compared with *rP298 lacZ* (Figure 2, A and A′).

The smallest fragment close to the start site, *duf −1.0 kb lacZ*, is capable of driving mesodermal expression in the developing embryo during stages 12–14 (Figure 2, B and B′). The reporter expression is seen in many somatic FCs in the abdomen. The pattern appears to be slightly diffuse. This reporter also ectopically marks a ventral cluster of cells in all the three thoracic segments (arrows, Figure 2, B and B′). This is in the region where adult imaginal myoblasts reside. No expression is seen in the developing visceral mesoderm. In a slightly larger fragment of the enhancer, *duf −1.5 kb lacZ*, the ectopic expression in the ventral thoracic region seen in *duf −1.0 kb lacZ* is completely repressed (Figure 2, C and C′). *duf −1.5 kb lacZ* also shows strong expression in ventral and dorsal clusters of somatic FCs in the abdominal segments (arrowheads, Figure 2, C and C′). Weak expression is also seen in the lateral cluster of FCs of the abdomen. There is no expression in any of the visceral muscles. *duf −2.4 kb lacZ* is expressed very weakly in all somatic founder myoblasts of the abdomen at stage 12–14 (Figure 2, D and D′). There is no expression in any of the visceral muscles. *duf −3.0 kb lacZ* is specifically expressed in longitudinal visceral muscle FCs that originate from the caudal mesoderm (arrow, Figure 2E). These FCs migrate from the posterior end over the developing embryonic viscera (gut) during stage 12. They fuse with the remaining FCMs in the gut region and align longitudinally along the entire length of the embryonic gut by stage 14 (arrow, Figure 2E′). *duf −3.0 kb lacZ* expression is also seen specifically in 3 FCs in the abdomen (arrowheads, Figure 2, E and E′). No expression is seen in circular visceral FCs or in any other somatic myoblasts. In comparison, *duf −3.8 kb lacZ* shows very specific expression in a large subset of somatic muscle FCs of the thorax and the abdomen. Expression is absent from circular and longitudinal visceral FCs. *duf −4.6 kb lacZ* (Supplementary data Figure S2) and *duf −5.1 kb lacZ* (Figure 2, G and G′) shows strong expression in a larger number of somatic muscle FCs and is again absent in visceral mesoderm founders. *duf −5.35 kb lacZ* and *duf −5.5 kb lacZ* (data not shown) also show strong expression in somatic muscle FCs very similar to *duf −5.1 kb lacZ* (Figure 2, G and G′) and very weak expression in the visceral mesoderm. Thus, from −3.8 to −5.5 kb, there is no change in the muscle type except that the number of somatic myoblasts expressing the reporter is increased. Preliminary analysis of the *duf* −6.4 kb fragment indicates that it is expressed weakly in somatic muscles and ectopically in the trachea. *duf −7.2 kb lacZ* reporter expression is seen in both somatic and visceral muscles as well as garland cells where wildtype *duf* is known to be expressed [4], but embryonic muscle expression is very weak compared to other reporter constructs (Supplementary data Figure S2).

Deletions from the proximal end i.e. enhancer constructs without sequences close to the start site, show a different pattern of reporter expression. *duf −8.2 −0.6 kb* (Figure 2, H and H′), *duf −7.9 −3.8 kb* (Supplementary data Figure S2) and *duf −9.8 −3.8 kb* (Figure 2, I and I′) fragments are specifically expressed in FCs of the developing mid-gut circular visceral muscles and also in a subset of somatic muscle FCs of the abdomen at embryonic stage 12–13. At stage 14, expression is clearly seen in the circular visceral muscles in a ribbon-like arrangement following fusion with neighboring FCMs (Figure 2H–I′, ). The expression is completely restricted to circular visceral founder cells and completely excluded from the longitudinal visceral muscle founders in the gut. The entire length of the putative enhancer *duf −9.8 kb lacZ* recapitulates the complete wildtype *duf* expression pattern in all the embryonic somatic as well as both types of visceral muscle founder cells (Figure 2, J and J′). Preliminary analysis of some of the smaller proximal and distal enhancer deletion reporter constructs, for example *duf −4.6−3.0 kb Gal4* showed nonspecific and ectopic reporter expression in the epidermis (Supplementary data Figure S2) while *duf −2.4−1.5 kb Gal4* had no detectable mesodermal expression pattern (data not shown). From this expression analysis it is clear that elements in the 10 kb region 5′ of *duf* are capable of driving reporter expression in different subsets of muscle FCs. This also indicates that there are independent modules for *duf* expression in different muscles. These modules are not noticeably overlapping, eliciting expression in a different subset of muscles with every addition of a few hundred base-pairs of the enhancer. Each additional fragment in the 5′ represses the expression seen in a smaller proximal fragment and directs expression in different domains of *duf* expression. All the necessary elements for this complex spatio-temporal regulation of *duf* expression in all embryonic muscle FCs appears to be located in the 10 kb region 5′ of *duf* coding region.

### Duf Enhancer Modules Mark Different Subsets of Embryonic Muscle Founder Cells

**Figure 3:**
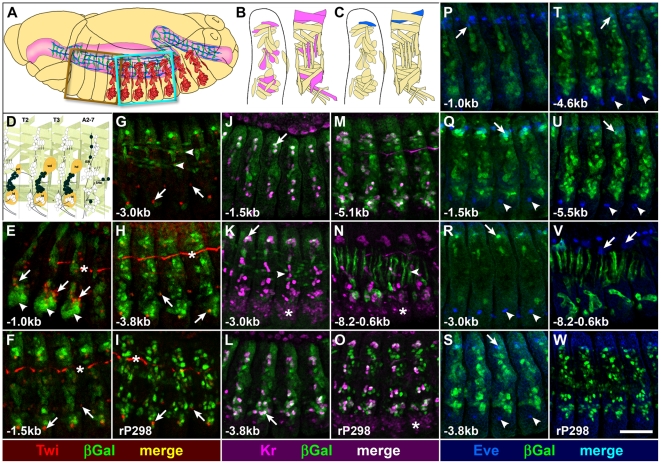
*duf* enhancer reporters are expressed in specific muscle founder cells. Cartoon representation of *Drosophila* embryo depicting different muscles (A) and Kr expression (in B) and Eve expressing DA1 (in C). (D) Brown box region (in A) is enlarged to show Twi expressing adult muscle precursors (AMPs, adapted from [74]). The same region is discussed in (E). (F–W) Confocal projections of stage 14 embryos double labeled with antibodies against β Gal (in green, E–W) and either Twi (in red; E–I), Kr (in magenta; J–O) or Eve (in blue; P–W) corresponding to cyan box region in (A). Wildtype *duf* expression is seen using *rP298 lacZ* (I, O and W). Ectopic expression of *duf −1.0 kb lacZ* (arrowheads in E) is ventral to Twi expressing AMPs (arrows in E). All *duf* enhancer reporters do not colocalize with Twi in the abdomen (arrows in F–I). Longitudinal visceral muscles are seen in *duf −3.0 kb lacZ* (arrowheads in G). Non-specific staining of trachea (asterisk in E, F and H). *duf −1.5 kb lacZ* colocalizes with Kr positive FCs (arrow J). *duf −3.0 kb lacZ* is expressed in longitudinal visceral muscles (arrowhead) and colocalizes with Kr only in DA1 (arrow in K). *duf* −*3.8 kb lacZ* (L) and *duf −5.1 kb lacZ* (M) colocalize with all Kr positive somatic FCs. *duf −8.2−0.6 kb Gal4* expressed in circular visceral muscles (arrowhead in N) do not colocalize with Kr. Kr expression in CNS is marked by asterisk. (P) *duf −1.0 kb lacZ* is not expressed in DA1 or pericardial cells. *duf −1.5 kb, −3.0 kb, −3.8 kb, −4.6 kb* and *−5.5 kb* colocalize with Eve in DA1 (arrows) but not with pericardial cells (Q–U). *duf* −*8.2−0.6 kb Gal4* is not expressed in DA1 (arrows) or pericardial cells (V). Eve expression in the CNS (arrowheads Q–U). Scale Bar  = 50 microns.

The expression pattern seen with *duf* upstream-lacZ reporters appears to be in specific subsets of FCs in different constructs. We double labeled enhancer-reporter constructs with mesodermal and founder- cell markers to verify this. Expression patterns of the *duf* enhancer lines were also confirmed by co-localization with *duf* Gal4 driven UAS-GFP (Supplementary data Figure S3). To examine if any *duf* upstream enhancer construct ectopically expresses in Twi positive adult muscle precursors or imaginal myoblasts in the embryo (see Figure 3D), stage 12–15 embryos of enhancer transgenic lines were double labeled with antibodies against β-Gal and Twi (Figure 3, E–I). *duf −1.0 kb lacZ* shows strong expression in the ventral thoracic region, where the imaginal disc primordia reside (Figure 3E). The expression pattern of the reporter appears to colocalize in this region with some Twi positive cells adhering to the imaginal discs (arrows Figure 3E). The reporter also ectopically marks some ectodermal cells (i.e. non-mesodermal cells) of the ventral imaginal disc primordia (arrowheads Figure 3E). *duf* −*1.0 kb lacZ* does not express in Twi positive adult muscle precursors of the abdomen. No other construct shows expression in any of the Twi positive adult precursors or imaginal myoblasts in the embryo. This indicates that the regulatory elements located −1.0 kb region immediately upstream of *duf* are sufficient to promote reporter expression in most of the embryonic mesoderm including the ventral thoracic region similar to Twi expression domain. Elements present further upstream as in *duf −1.5 kb lacZ* have repressor elements that suppress expression in ventral thoracic region but at the same time also promote the expression of the reporter in the dorsal and ventral subset of somatic FCs recapitulating a part of wildtype *duf* expression.

Additionally, co-localization was done with the *duf* enhancer reporter constructs with other known FC markers such as Kruppel (Kr), Vg and even-skipped (Eve). These transcription factors mark different somatic muscles as follows: Kr is expressed in DA1, DO1, LL1, LT2, LT4, VL3, VA2, VO2 and VO4 somatic FCs (Figure 3B). Vg is expressed in DA1-3, LL1, VL1, VL2, VL3, VL4 somatic muscle FCs and Eve is expressed only in DA1 muscle founder (Figure 3B) and a subset of pericardial cells. Colocalization with Kr (Figure 3, J–O), Eve (Figure 3, P–W) and Vg (data not shown), reveal that the expression pattern of the reporter constructs is in specific founders. Co-localizations with *duf* Gal4 > UAS-GFP (Supplementary data Figure S3) and Kr were also very useful in identifying specific muscles that were marked by different *duf* enhancer constructs. *duf −1.5 kb lacZ* is expressed in DA1 and DO1, and very weakly in other Kr positive FCs. *duf −3.0 kb lacZ* is expressed only in DA1 among all Kr expressing FCs. *duf −3.8 kb lacZ* is expressed strongly in 6 Kr positive somatic FCs but very weakly in LT2, LT4 and VO2 somatic FCs. In *duf −3.8 kb Gal4* no expression was detected in LT2, LT4 and VO2 somatic FCs but there was strong expression in other Kr positive somatic FCs. *duf −4.6 kb lacZ* (data not shown) and *duf −5.1 kb lacZ* are expressed weakly in LT2, not detectable in VA2 but strongly in all other Kr positive somatic FCs. *duf −8.2−0.6 kb* Gal4 is expressed weakly in VO4 but in none of the other Kr positive FCs. *rP298 lacZ* was used as positive control for comparison. Colocalization of all *duf* enhancer constructs with Eve also revealed that none of the enhancer reporters tested expressed ectopically in pericardial cells (Figure 3, P–W). Eve is expressed in DA1 somatic FC and subset of pericardial cells in the dorsal mesoderm. *duf −1.0 kb lacZ* (Figure 3P) is expressed the dorsal row of cells which are not pericardial cells. No colocalization is detected in pericardial cells. *duf −1.5 kb, −3.0 kb, −3.8 kb, −4.6 kb* and *−5.5 kb* enhancer lacZ lines colocalize with Eve expressing DA1 somatic FC but not with pericardial cells (Figure 3, Q–U). *duf −8.2−0.6 kb Gal4* is not expressed in Eve positive DA1 or pericardial cells (Figure 3V).

Interestingly, in two cases, *duf* enhancer reporters are expressed in domains where wildtype *duf* expression is not known. These expression patterns are described in Supplementary data Figure S4. By stage 16, *duf −2.4 kb lacZ* is also expressed ectopically in a large subset of the developing cardioblasts, those that express *seven-up (svp)* but not *tin*. Wildtype *duf* is not expressed in the developing cardioblasts and loss of *duf* function does not affect the formation of the embryonic heart [4]. The cardioblast expression seen in *duf −2.4 kb lacZ* is completely repressed in a slightly larger *duf* enhancer fragment: *duf −3.0 kb lacZ*. Similarly, the reporter expression in *duf −3.0 kb lacZ* is also seen in the embryonic central nervous system in a large subset of neuroblasts as compared to wildtype *duf* at stage 16. All the larger *duf* enhancer constructs do not show ectopic reporter expression in the ventral thoracic segments, cardioblasts or embryonic central nervous system. This suggests the presence of repressor elements that would restrict *duf* expression specifically to different founder cells.

### Distal Enhancers Regulate *duf* Expression During Adult Myogenesis

**Figure 4:**
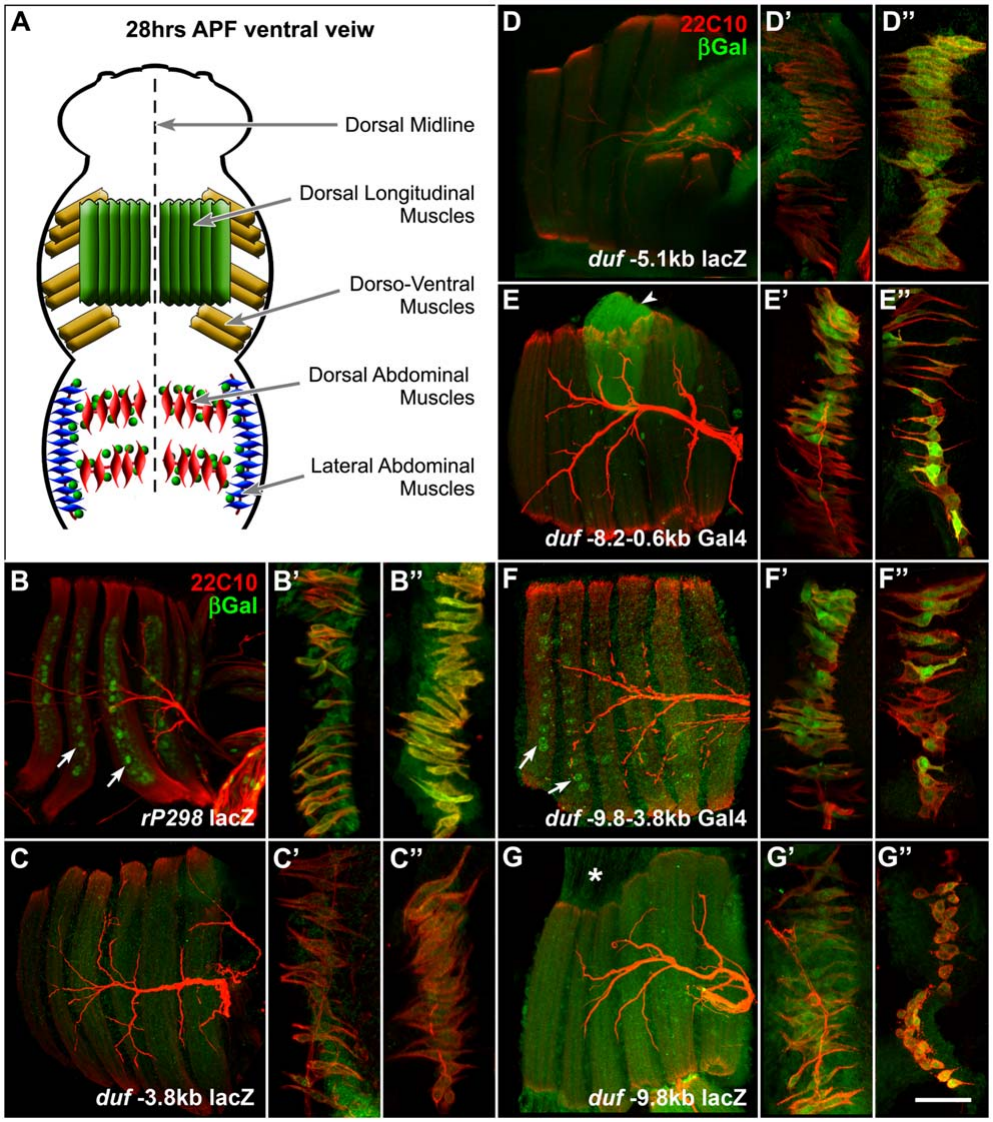
Expression of *duf* enhancers in developing *Drosophila* adult muscles. (A) Diagrammatic representation of 28 hr APF (after puparium formation) pupa depicting developing muscles of the thorax and the abdomen. Dorsal Longitudinal Muscles (DLMs; in green) Dorso-Ventral Muscles (DVMs; in yellow) make up the indirect flight muscles (IFMs) of the thorax. Abdominal dorsal muscles (red) and lateral muscles (blue) are shown (B–G″). Confocal projections of 28 (±1) hr APF pupae from *duf* enhancer lacZ lines double labelled with anti-β-gal (green) and m22C10 (red). Pupal thoracic DLMs (six fibres, B–G), abdominal dorsal muscles (of a2 hemisegment, B′–G′) and lateral muscles (B″–G″) are shown. *rP298 lacZ* expression is seen in the nuclei of six DLM fibers (B) and *duf Gal4* driven lacZ is seen in of dorsal (B′) and lateral muscle FCs that are also marked by m22C10 (B″). *duf −3.8 kb lacZ* expression is weak in DLMs (C) and dorsal muscles (C′) but stronger in lateral abdominal muscles (C″). *duf −5.1 kb lacZ* is expressed in lateral muscles (D″) but not in DLMs (D) or dorsal muscles (D′). *−8.2 to −0.6 kb Gal4* drives expression in DVMs. DVM II is seen here (arrowhead in E). Specific expression is seen in dorsal muscles (E′) and lateral muscles (E″). Some amount of variation is seen in the expression of this Gal4. *duf −9.8−3.8 kb Gal4* is specifically expressed in DLMs (arrows in F). It also drives expression in dorsal (F′) and lateral muscles (F″). *duf −9.8 kb lacZ* is expressed in DLMs (G) and DVMs (data not shown) and in lateral muscles (G″) but less specifically in the dorsal muscles (G′). Background expression is also seen in FCM population and attachment fibers (asterisk in G). Anterior to the top, dorsal midline to the right (B–G and B″–G″); and anterior to the right, dorsal midline to the top (B′–G′). Scale bar = 50 µm.

Adult muscle founder-specific expression of all the reporters was assayed in imaginal myoblasts associated with the wing imaginal discs (data not shown) and during pupal myogenesis for all the different muscle subtypes in the thorax, and Dorsal, Ventral and Lateral muscles in the abdomen. Expression was compared by colocalization with mouse monoclonal antibody 22C10, which marks neurons and the abdominal founder myoblasts very clearly [11]. *rP298 lacZ* or *duf Gal4-UAS lacZ* was used for wildtype comparison (Figure 4).

Analysis of *duf* enhancer deletion constructs during different stages of adult myogenesis reveals several interesting aspects of *duf* regulation in the adult. All smaller *duf* enhancer constructs up to 3.8 kb show no, or comparable to background, reporter expression in both thoracic as well as abdominal muscle founder cells of the adult. *duf −3.8 kb lacZ* shows weak expression in lateral abdominal muscles (Figure 4C″). *duf −5.1 kb* is clearly expressed in lateral abdominal muscles but not in dorsal abdominal muscles (Figure 4D″). This expression is not seen in a slightly larger fragment i.e. *duf −5.5 kb lacZ* (data not shown) which indicates that region around −3.0 −5.1 kb upstream of *duf* has a module for expression in lateral abdominal muscles. None of the constructs up to those with −5.5 kb of 5′ sequence are expressed in dorsal abdominal muscles or in any of the thoracic muscles. *duf −8.2−0.6 Gal4* is expressed strongly in a subset of dorsal as well as lateral abdominal muscles (Figure 4, E′ and E″). Expression is also seen in one kind of indirect flight muscle of the thorax –the Dorso-Ventral Muscles (DVMs) (Figure 4E). There is some variation in the expression pattern of this particular construct. The *duf −9.8 −3.8 kb Gal4* is specifically expressed in all adult muscle founders. It marks the founder larval templates [11], [42] of the Dorsal Longitudinal Muscles (DLMs) in the thorax (Figure 4F) and dorsal, lateral and ventral abdominal founders [14] (Figure 4, F′ and F″). The larger *duf −9.8 kb lacZ* shows expression in all the developing adult muscle founders (Figure 4, G–G″) and also marks several epidermal cells ectopically.

The results show that enhancer fragments close to the *duf* transcription start site are very important to drive expression in specific muscles of the embryo and deletion of these elements appears to enhance expression specifically in the adult muscles. Taken together, these results merited further computational analysis of this region.

### Computational Study of the *duf* Enhancer

The complex and non cumulative expression pattern observed in *duf* enhancer reporter constructs justified a more detailed study that we describe below, where we made use of Stubb and PhyloGibbs-MP [31], an updated version of the motif-finder PhyloGibbs [29] that is capable of module prediction either *ab initio* or using prior position weight matrices (PWMs). First, we made a list of high-quality PWMs for factors of known importance in mesoderm development, using sequences from PWMs, using the Flyreg database of DNAse I footprints [32], binding sequences found in recent bacteria-one-hybrid system (B1H) studies [33], [43], and weight matrices from the literature. In all, 44 matrices were generated in this way, including six from the literature, 11 from the B1H data Noyes *et al.* [33], [43], and the remainder from the Flyreg database of DNAse I footprints [32]. The details are described in Materials and Methods. These high quality PWMs and their associated sequence logos are in Supplementary data Figure S5. A subset of 31 matrices were eventually used, the remainder being poor-quality (not specific enough) or not making significant predictions in preliminary runs.

Next, we used the module prediction program Stubb [19], [20] with these PWMs to predict enhancers upstream of and in the intron of *duf*. Finally, we used the same PWMs and PhyloGibbs-MP to confirm the prediction of the enhancers as well as to predict individual binding sites for transcription factors.

### Predicting Enhancers with Stubb

We used the 31 PWMs described above with Stubb [19], [20] to determine upstream and intronic regions of interest. Unlike naive methods based on clustering predicted sites, Stubb incorporates competition between factors, carefully handling the situation where multiple factors may compete for the same binding sequence; it calculates a “partition function” that takes account of all possible ways of “parsing” a sequence into regulatory sites and “background”, and then uses this to predict “binding energies” for each factor at each site. It also calculates an overall “free energy” function that serves to indicate likely locations of CRMs.

**Figure 5:**
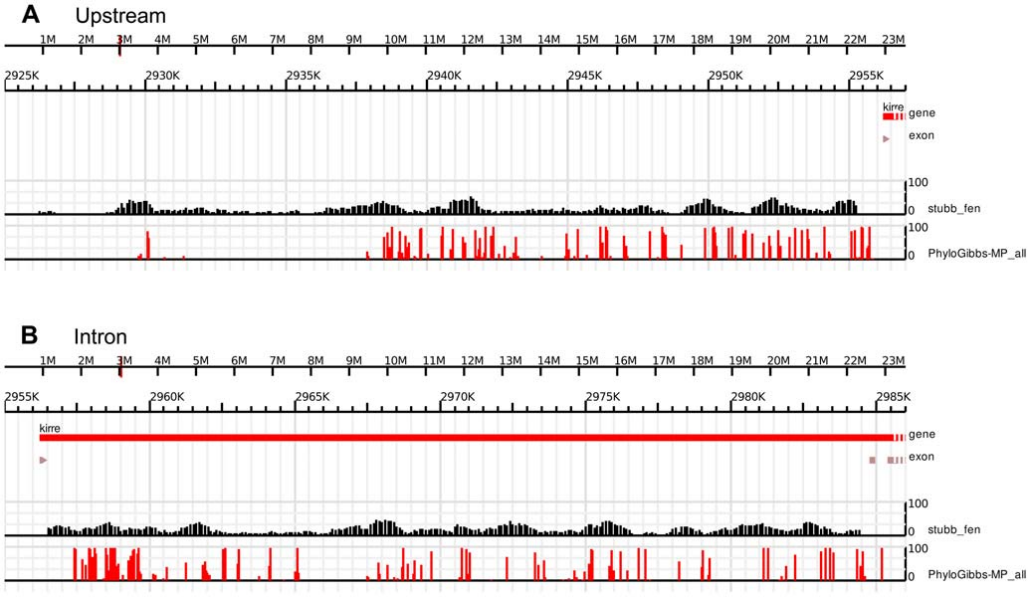
Stubb and PhyloGibbs-MP module predictions for *duf* genomic region. Using 31 position weight matrices constructed from the FlyReg database, from B1H data (Noyes et al.), and from other sources, for mesoderm-relevant transcription factors, Stubb predicts a modular structure both in the upstream and in the intronic region. PhyloGibbs-MP, run in module-prediction mode, predicts a similar modular structure. In particular, much of the first 20 kb upstream of *duf* appears to be potentially regulatory, with significant modular structure in the first 10 kb upstream and again 15–18 kb upstream of the transcriptional start site. Another enhancer appears about 25 kb upstream of the transcriptional start, which is only weakly picked up by PhyloGibbs-MP, suggesting that it may bind a different set of factors. Both programs pick up significant modules at both ends of the intron and some isolated clusters in the middle.

We ran Stubb on the 30 kb upstream region of *duf*, and also on its 29 kb intron, using a window size of 1000 bp and a shift of 100 bp. The free-energy profile shows significant enhancer structure: the first 10 kb upstream have significant binding free energies for these factors, but there also occur peaks at about 12 kb −15 kb upstream and 25 kb upstream (Figure 5A), and in the intron (Figure 5B).

Apart from global free energies, Stubb predicts binding affinities for individual factors at individual sites, taking competition with other factors into account. Some factors show numerous low-affinity sites, usually attributable to a poorly-defined input weight matrix. Some factors, however, seem to show distinct clustering in certain parts of the 10 kb region. A discussion of binding site predictions for individual factors is deferred to the next subsection.

### Predicting Enhancers and Regulatory Sites with PhyloGibbs-MP

PhyloGibbs is a motif-finder with the ability to incorporate orthologous sequence from closely-related species that may have significant non-functional conservation. It reports predictions with significance estimates that are posterior probabilities, obtained from extended sampling, that the predictions are binding sites given the prior assumptions. We showed [29] that these significance estimates are reliable in synthetic data, and given some reasonable assumptions on gene regulation in yeast (*Saccharomyces cerevisiae*) and the state of present experimental knowledge, the significance estimates are probably very accurate in experimental systems too.

PhyloGibbs-MP is an update to PhyloGibbs, which, among other new features, has the ability to localise predictions to small regions with a specified size and average spacing, meant to represent CRMs. Its predictions of CRMs here mainly agree with Stubb′s. However, its main task is to predict binding sites, either *ab initio* or with the help of “informative priors” such as our 31 mesoderm-relevant position weight matrices. While Stubb uses PWMs directly to predict sites, PhyloGibbs-MP seeks sites that are similar to one another, and only uses the PWMs to bias the search (in the form of a Bayesian prior). Thus, predictions differ between the two programs: PhyloGibbs-MP will fare poorly when only very few copies of a site are present, but will pick up more numerous sites even if they correspond relatively poorly to the known PWMs, or do not correspond to the supplied PWMs at all. It will, however, report a match to a known PWM only if the match is significant, that is, the PWM generated by the PhyloGibbs-MP alignment corresponds sufficiently closely with the prior PWM.

**Figure 6:**
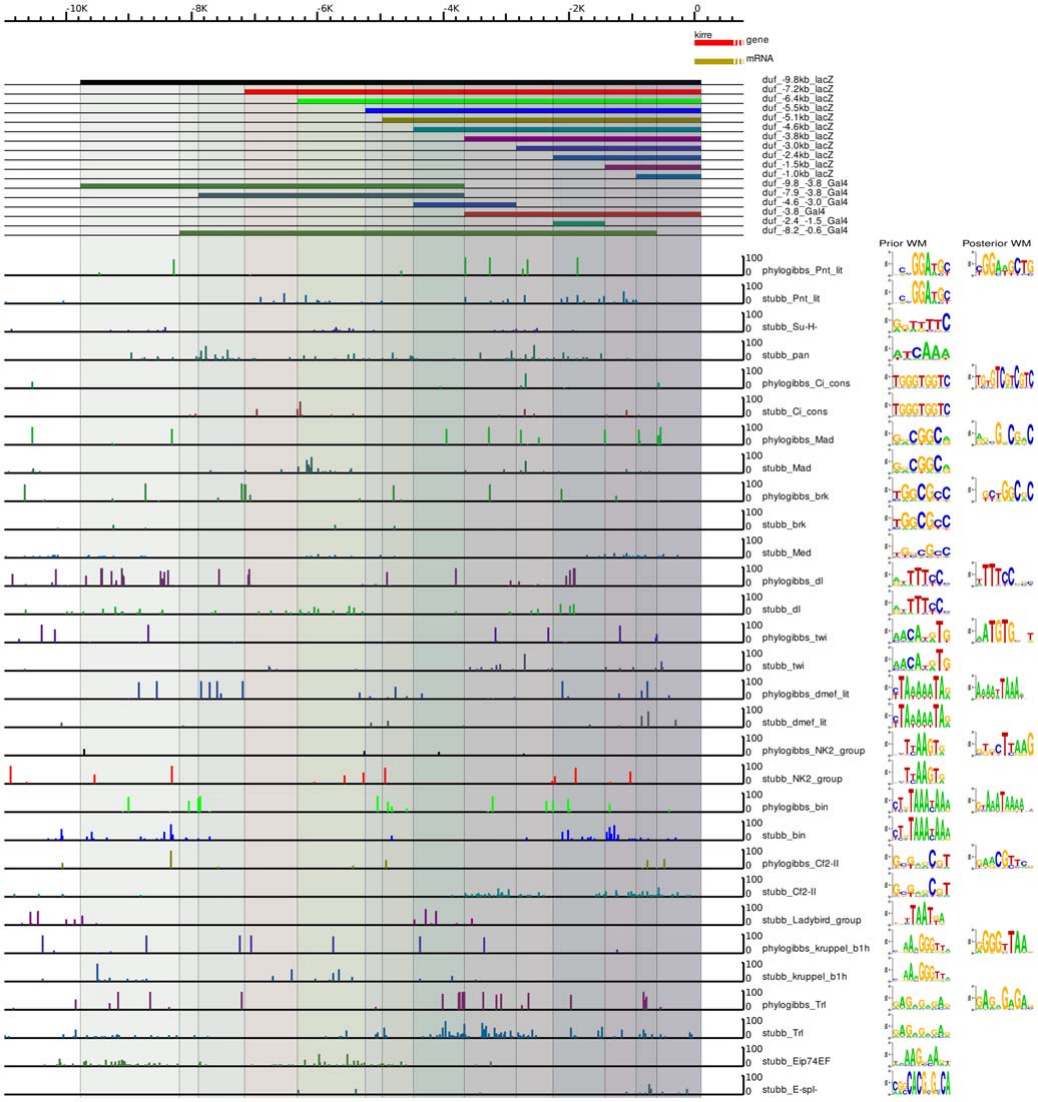
PhyloGibbs-MP and Stubb results for 11 kb *duf* upstream. Predictions of individual binding sites, for various factors of interest, from Stubb and PhyloGibbs-MP along with different *duf* enhancer reporter deletion constructs. Stubb predicts a binding affinity of a known factor using its previously characterised position weight matrix, while PhyloGibbs-MP predicts sites that are similar to one another, but allows its search to be biased via “informative prior” weight matrices. For both programs, position weight matrices for 31 mesoderm-relevant factors, as discussed in the text, were used as priors. These are shown in the left column of sequence logos, marked “Prior WM”. PhyloGibbs-MP in addition reports a posterior WM for all motifs it finds, which is a base-count of all sites reported in each motif, weighted by their significance. Sequence logos for these are in the right column, “Posterior WM”. Stubb reports no posterior WM. PhyloGibbs-MP′s posterior WMs are, in general, similar to the prior WMs, at least in their core features. Moreover, several positions in each PWM have high information scores, indicating a high degree of similarity among the predicted sites. These two facts encourage confidence in the quality of PhyloGibbs-MP′s site predictions as well as in the associations with the prior WMs. Nevertheless, as with any bioinformatics program, some false positives are expected, and several genuine sites may have been missed. The sequence logos were made with Weblogo 2.8 [75]. The predictions were plotted with our genome visualisation tool (S. Acharya and R. Siddharthan, unpublished).

We ran PhyloGibbs-MP in module-prediction mode (specifying that about half the input sequence is expected to be functional) on the 30 kb upstream of *duf*, and also on the intron, and found a similar modular structure as with Stubb (Figure 5). The figure shows free energies for Stubb, and predictions of individual binding sites for PhyloGibbs-MP. The binding sites predicted by PhyloGibbs-MP generally cluster within the peaks of the free energy calculated by Stubb. We did additional PhyloGibbs-MP runs on the first 11 kb upstream of *duf*, specifying that all of this region is expected to be functional, and also specifying two possible widths of binding site motifs (9 bp and 12 bp). We find predicted binding sites for several key factors (Figure 6). Predictions for the second set of homeo domain factors with some relevance to mesoderm development are shown in Supplementary data Figure S6. By and large the above predictions agree with those from Stubb, which was run using the same weight matrices, and match several published consensus sites (see Supplementary Datasheet S3). Differences in predictions are expected because Stubb reports matches to a prior factor, but does not consider the similarity of different matches to one another; whereas PhyloGibbs-MP reports conserved motifs, scored on the similarity to one another of different sites associated with each motif, and gives secondary importance to a match with a prior PWM. Nevertheless, the two programs make largely similar predictions. If we consider predictions in the key sequence window 11 kb upstream of *duf*, of 179 site predictions made by PhyloGibbs-MP with a significance of 0.1 or more, 138 share a 4 bp or better overlap with predictions made by Stubb (with a significance of 0.1 or more). (Stubb makes many more predictions at a given significance, however.) While some factors have sites scattered throughout the enhancer, others appear to be localised to certain regions of the enhancer. Most significant predictions by Stubb and Phylogibbs (Figure 6) are in good concordance with the observed expression data of the *duf* enhancers: some such examples are discussed below.

Integration of major signaling networks such as Dpp, Hh, Wg, Notch and Ras with mesodermal transcription factors is very important for specification of the mesoderm and development of different muscle types (reviewed in [1], [44]). In reporter expression studies, the proximal enhancers show very diverse expression in different muscle types of the *Drosophila* embryo. This includes dorsal and ventral group of somatic founders, ectopic expression in cardioblasts, longitudinal visceral founders and large subset of somatic founders. No significant expression is detected in any of the adult muscles in constructs up to *duf −3.8 kb lacZ*. In this region, among the signaling pathway effectors, strong Pnt binding sites are predicted by both PhyloGibbs and Stubb Weak Su(H) and Pan (dTCF) sites are predicted by Stubb. Phylogibbs predicts strong Mad binding sites and few Brk binding sites in the proximal constructs. Both programs predict couple of Ci binding sites in the −3.0 kb construct (Figure 6).

Several clusters of binding sites are also predicted for TFs that are important for mesoderm development (Figure 6). Twi, which is critical for mesoderm specification, has two clusters predicted by both programs in constructs −1.0 and −1.5 kb and another in constructs −3.0 kb and −3.8 kb. Similar structure is also seen for *Drosophila* zinc finger transcription CF2-II, a myogenic marker downstream of MEF2 during muscle development. The somatic-visceral subdivision of the embryonic mesoderm is initiated by Dl gradient thresholds [45]. Several Dl binding sites are predicted between −2.0 to −4.0 kb. dMef2 being another master regulator of myogenic differentiation also has clusters predicted in constructs −1.0 kb (both PhyloGibbs and Stubb), −1.5 kb and −2.4 kb (PhyloGibbs). These predictions agree very well with pan mesodermal expression seen in smaller constructs (*duf −1.0 kb lacZ*) that gets restricted more specifically to somatic muscles FCs in *duf −3.8 kb lacZ*.

Bin and NK2 group of HD factors are known to be critical for visceral mesoderm development. Stubb and PhyloGibbs predicts clusters of binding sites for bin and NK2 in constructs −1.5, −2.4 and −3.0 kb and just one site in −*3.8 kb lacZ* (Figure 6). This agrees very well with the predominant visceral muscle expression of *−2.4 kb* and −*3.0 kb lacZ* constructs (Figure 2). Additionally, Stubb and PhyloGibbs predicts clusters of binding sites for bin and NK2 in −7.8 to −9.8 kb region. *duf −9.8−3.8 kb* and *8.2−0.6 kb* enhancers constructs are also expressed specifically in circular visceral muscles and few somatic muscles of the embryo. PhyloGibbs also predicts one twi site and cluster of dMef2 sites in this region.

More distal *duf* enhancer constructs (*−5.5 kb* to *−9.8 kb lacZ*) drive expression in adult specific muscle FC cells. Stubb predicts two clusters of Ecdysone-induced protein 74EF (Eip74EF) binding sites in *duf* enhancer. One cluster is in constructs −5.5 kb and −6.4 kb and a second cluster is constructs −8.6 kb and −9.8 kb. Stubb also predicts another Pnt, Pan, Mad and Ci cluster between −5.5 kb to −8.6 kb region. Stubb predicts ladybird group binding sites in 5.5 kb lacZ. dMef2 cluster is predicted by both programs in the 5.5 kb construct and another cluster in the distal region between −7.2 kb to −9.5 kb (by PhyloGibbs). This region shows distinct enhancer activity during adult myogenesis. STAT binding sites are found in two clusters, one cluster in proximal enhancers, which show embryonic expression and second cluster in distal enhancers, which show adult expression (Supplementary data Figure S6). Antp and Abd-B group factors have fewer sites in proximal enhancers and several sites predominantly in the distal enhancers (Supplementary data Figure S6).

**Figure 7:**
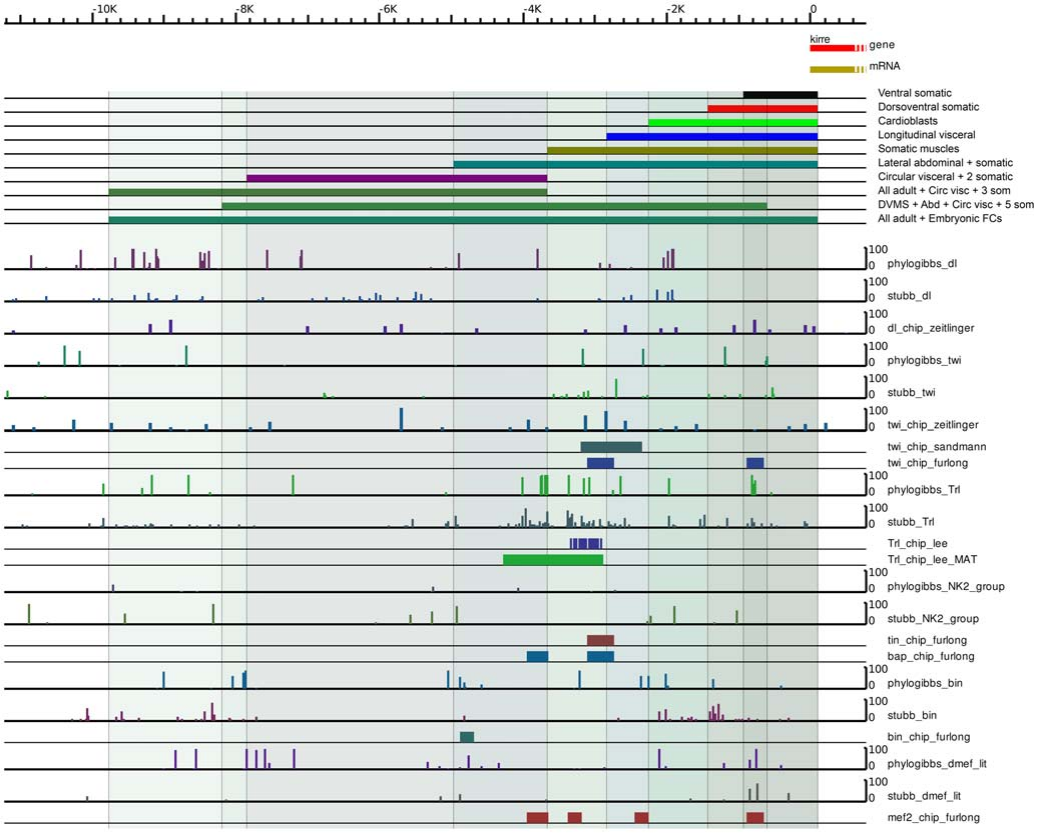
Comparison of PhyloGibbs-MP and Stubb predictions with ChIP data. Comparison of PhyloGibbs-MP and Stubb predictions with ChIP data where available in the literature. Expression pattern of different *duf* enhancer reporter deletion constructs are also shown. The graphs “dl_chip_zeitlinger” and “twi_chip_zeitlinger” are plots of regions with reported MedianOfRatios >1.0 (logs of ratios are plotted) from the file MoRs.xls in the supporting data of Zeitlinger et al. While the original authors report no significant binding by the factors dorsal or twist in this region, these less significant predictions show correlation with our predicted binding sites, and in the case of twist, with ChIP data from elsewhere. The graph “twi_chip_sandmann” is from the supporting data of Sandmann et al. The graph “Trl_chip_Lee” is from Lee et al [47]. The graph “Trl_chip_Lee_MAT” is a reanalysis of the same data from Lee et al, using the program MAT [48], as described in the text. The graphs labelled “_furlong” are unpublished data from the Furlong Laboratory, EMBL, Heidelberg (E Furlong, personal communication). In most cases, ChIP predictions corroborate significant binding region predictions via our computational approaches. It should also be noted that most of these ChIP experiments were done over early development time courses, and therefore may not adequately reflect binding during myoblast development.

Additionally, ChIP data are available for the following factors: Dl, Twi, and Sna [36]; Mef2 [34]; Twi, Mef2, Tin, and Dl [35]; Bap and Bin [46]; and Trl [47], and we compared our predictions with these data. Much of these data are for early embryonic development stages and suggest no significant binding near *duf*; however, we find striking agreement with our predictions with published data for Trl [45] and Twi [34] and with more recent data for Tin, Mef2, Bap and Bin [unpublished data from the Furlong Laboratory, EMBL, Heidelberg; E. Furlong, personal communication; see Figure 7]. In the case of Dl [35], no significant predictions are made by the authors, but a plot of lower-significance predictions from the supplementary data of the same paper agrees well with our predictions. Similarly, while Twi binding is predicted via ChIP in [34], there are no significant predictions in [35]; but again lower-significance predictions from the supplementary data agree with our predictions as well as with the ChIP results from [34]. Finally, the published ChIP predictions for Trl [47] support our predictions; a re-analysis of the raw tiling array data using MAT [48], with a specified *p*-value of 0.05, results in a larger region that strikingly overlaps our predicted sites. These data along with expression domains of duf enhancer modules are shown in Figure 7.

## Discussion

### Gene Regulation in *Drosophila* Muscle Founder Cells

A gene which is expressed in all cells of one category—*duf* in muscle FCs, for example—can be proposed to be regulated by a relatively simple mechanism, which is a consequence of the specification of that broad cell type. In fact, from earlier studies on mesodermal enhancers of *eve*, a network of factors - dTCF, Mad, Pnt, Twi and Tin - have been shown to positively and negatively regulate the specification of muscle FCs and also FCMs [13]. Further, by using specific genetic perturbations, several genes with localized expression in FCs and FCMs were identified [49]. Philippakis et al. [50] attempted to identify enhancers that contained matches to five transcription factor binding site motifs– dTCF/Mad/Pnt/Twi/Tin—as generalized regulators of FC gene expression, which identified a *heartbroken (hbr)* enhancer that drove expression in dorsal FCs- indicating that the FC gene regulation was not exclusively regulated by these five TFs. But with a smaller and more general subset of TFs i.e. Pnt, Twi, and Tin, four new enhancers that drive FC like expression were identified [50]. We suspect that FC gene regulation is more complex than previously proposed. Additional mechanisms and combinations of dTCF/Mad/Pnt/Twi/Tin along with other, currently unknown, motifs may regulate FC gene expression. Our results from the analysis of *duf* regulation during embryonic and adult myogenesis also reflects this complexity. They demonstrate the complex regulation of a gene that is expressed in all founder cells. Spatio-temporally regulated activation/repression of specific *duf* enhancer modules in distinct muscle types suggests an elegant mechanism of generating muscle diversity by transcriptional control of a key player in myoblast fusion.

### Functional and Experimental Evidence for *duf* Enhancers

The smallest enhancer fragment, *duf* −1.0 kb, close to the transcriptional start site has basic elements that accurately identify mesodermal lineage (Twi, see also Figure 7) and allows expression in some of the somatic muscles. In slightly larger constructs (for example: *duf* −1.5 kb, −3.0 kb and −3.8 kb), the reporter expression is very well restricted to specific muscle types. Several ChIP-on-chip results indicate Twi binds between −2.4 kb to −3.8 kb of the *duf* enhancer (Figure 7). In this region, we also find sites for Pnt, Mad and DMef2 that match the published consensus. ChIP-on-chip studies (Figure 7) indicate these Dmef2 sites are occupied during early mesodermal development. DMef-2 is a downstream target for *twi*, its early expression pattern modulates as the mesoderm organizes into cell groupings with distinct fates. DMef2 is expressed in the segregating primordia as well as the differentiated cells of the somatic, visceral and heart musculature. We also find Nk2 group (Tin, bap) sites in the −1.5 to −2.4 kb using Stubb. When this fragment (*duf −2.4 to −1.5 kb*) was analyzed independently, it was found to be insufficient to drive any reporter expression. Interestingly, *duf −2.4 kb lacZ* is expressed strongly in cardioblasts and very weakly in all somatic muscles, while the smaller *duf* −1.5 kb construct is expressed in large subset of somatic muscle FCs.

RTK signalling and Twi are critical for the somatic muscle fate [1]. ChIP-on-chip [34], [35] results show that binding of Twi (−3 kb ) and Dmef2 (−3 to −4 kb region) during early mesodermal development. These results corroborate our bioinformatic predictions and enhancer deletion studies. In addition, we predict Dmef2 binding sites in the −5 to −5.5 kb region.*duf −3.8 kb lacZ* and *duf −5.1 kb lacZ* are expressed strongly in majority of the somatic muscle FCs but not in any of the gut muscles. *duf −5.1 kb lacZ* is also expressed clearly in adult lateral abdominal muscles which is not seen in *duf −5.3 kb lacZ* and *duf* −*5.5 kb lacZ*.

### Enhancer Elements for Embryonic Gut and Body Wall Muscles

Body wall (somatic) muscles provide the force for the peristaltic locomotion of the larva while the gut (visceral) muscles provide the peristaltic force for movement of food during digestion. Longitudinal and circular muscles of the midgut as well as the visceral muscles of the foregut and hindgut arise from different primordia and follow diverse developmental pathways [51]. In contrast to most other larval tissues that are histolyzed during metamorphosis, the visceral musculature persists through metamorphosis. This might be an important aspect, as in our deletion studies we find *duf* enhancer fragments (*−9.8 to −3.8 kb, −8.6 to −0.6 kb*) express strongly in visceral muscles of the embryo and also in the persisitent larval muscles, which are FCs of the DLMs, at the onset of adult development.

Visceral mesoderm development is abnormal in *shn* mutants: *shn* mediates action of *Dpp* on mesodermal cells by inducing *bap* [52]. Phylogibbs finds couple weak Shn binding sites in this region (Supplementary data Figure S6). A stronger Shn binding sites are predicted further upstream by Stubb and Phylogibbs. Stubb predicts NK2 (which includes bap) binding sites in −1.5 kb to −2.4 kb constructs. PhyloGibbs predicts two weak binding sites in −2.4 kb and −3.0 kb constructs. New ChIP-on-Chip experiments (Furlong lab, personal communication) suggest that Bap binds in this region of *duf* enhancer in mesodermal cells from stage 6–8 of embryonic development (Figure 7). *duf −3.0 kb lacZ* is expressed strongly in Longitudinal Visceral Muscle FCs (Figure 2), that originate from the caudal mesoderm [53] but not in circular visceral FCs that arise from the midgut [54]. Similarly, several Bin binding sites are predicted by both Stubb and PhyloGibbs between −1.0 to −3.0 kb (Figure 6). *bin* is important for maintaining the distinction between visceral and somatic mesoderm and its activity is essential for differentiation of the visceral mesoderm into midgut musculature [55].

Deletions from the proximal end such as *duf −7.9−3.8 kb Gal4*, *duf −9.8−3.8 kb Gal4* and *duf −8.2−0.6 kb Gal4* all show consistent and strong expression patterns in circular visceral muscle FCs and very few somatic FCs in the embryo. Many visceral mesoderm factor binding sites are predicted in this region. For example, several NK2 group sites are predicted by Stubb and few weak sites are predicted by PhyloGibbs between −9.8 to −3.8 kb region. Again several Bin sites are predicted by Stubb and PhyloGibbs between −9.8 to −7.8 kb. Similarly, two clusters of byn sites are predicted by Stubb (Supplementary data Figure S6) between −1.0 to −3.0 kb and −5.5 to −7.2 kb.

Thus, the results from our bioinformatics analysis are in very good agreement with the transgenic deletion studies, ChIP-on-chip data and the existing literature.

### Enhancer Elements for Embryonic and Adult Myogenesis

*Drosophila* uses its genome to make two distinct developmental body plans: the larva and the adult. Duf is critical for myoblast fusion during both embryonic [4] and adult myogenesis [11], [14]. Embryonic muscles of *Drosophila* are single fibres whereas adult muscles are bundles of muscle fibres - similar to those of vertebrates [11], [14], [56], [57]. We find specific enhancer elements repsonsible for *duf* expression during adult myogenesis. *duf −9.8−3.8 kb Gal4* and *duf* −*8.2 −0.6 kb Gal4* are expressed strongly in all the adult muscle FC analogs during adult myogenesis. Removal of enhancer elements close to the transcription start site appears to promote expression in circular visceral muscles of the embryo and all adult muscles.

Ecdysone-induced protein 74EF (Eip74EF), has putative binding sites in the region >5 kb from the start site. PhyloGibbs predictes dMef2 sites between −7.2 to −9.5 kb (Figure 6). Several signaling pathway effectors also have distinct set of binding sites occuring in the proximal and distal regions of the enhancer (Figure 6). Several homeodomain factors (for example: Antp and Abd-B) also have several binding sites, predicted by both Stubb and PhyloGibbs predominantly in the distal region of the enhancer (Supplementary data Figure S6).

Addtionally, putative binding sites for GAGA factor encoded by the Trithorax-like gene (Trl) characteristic of TREs (Trithorax Response Elements) are found in −4.0 kb from the *duf* start site (Figure 1). Sites further upstream are predicted by Stubb and PhyloGibbs, but the strongest sites occur below −4.0 kb (Figure 6). This region shows good embryonic expression but no expression in adult muscles. Published ChIP predictions for Trl [47] agree well with our predictions in this region. A re-analysis of the raw tiling array data from Lee et al [47] using MAT [48] and a lower threshold, results in a larger region that overlaps a significant region of our predictions (Figure 7).

Similarly, putative binding sites for PHO (pleiohomeotic) and PHO-like polycomb group proteins (PcG) that bind to PREs (Polycomb group Response Elements) are found between −8.0 kb to −9.3 kb region (Figure 1). ChIP-on-chip experiments for TREs and PREs by Schwartz et al [58] find GAF (Trl) binding upstream of *duf*, but only very little binding of PHO, in Sg4 tissue culture cells. However, data from similar experiments using *Drosophila* embryos suggest that PHOL binds upstream of the *duf* promoter. PcG protein binding at *duf* has not been detected, but a binding peak for ASH1 is usually associated with PcG target genes when they are derepressed, has been found (Vincenzo Pirrotta, personal communication).

PREs and TREs in *duf* enhancer region appears restricted to specific regions. TRE/PRE mediated silencing may be responsible for switching between embryonic and adult specific enhancers by restricting access of TF to chromatin in appropriate tissue and time points. Experiments designed to test the presence or absence of each of these factors during embryonic or adult myogenesis would help answer this question more accurately.

Taken together, this study has uncovered a complex regulatory mechanism operating to control important myoblast fusion gene such as *duf* during *Drosophila* myogenesis. A set of enhancer constructs generated for this study will be valuable reagents in identifying important *trans-*acting factor-binding sites and chromatin regulation during myoblast fusion. We describe several contexts where the bioinformatics predictive tools and ChIP-on-chip approaches have great value and others where, clearly, more information is needed before predictive tools can be applied.

## Materials and Methods

### DNA constructs

Specific primers were designed to PCR upstream fragments of *duf* with *BamHI* or *EcoRI* overhangs to assist cloning. Details of the primers used and genomic location they cover are tabulated in Supplementary data Table S1. PCR products were cloned either into pCaSpeR AUG βGal [39], or pPTGal [40]. Sequence confirmed clones were used to generate transgenic flies. Reporter gene expression was assayed in a minimum of 5 independent insertion lines for every construct.

### Fly stocks

The following fly strains were used: *UAS lacZ* and *UAS GFP* stocks were from Bloomington Stock centre. *rP298 (duf) lacZ* [4], [41] and *duf Gal4* [16] with P insertion into the *duf* locus faithfully reproduces the wildtype *duf* expression pattern in muscle FCs. All transgenic *duf* enhancer-reporter deletion lines described were generated for this study.

### Immunohistochemistry

Anti-β-galactosidase antibodies raised in Rabbit (Molecular Probes) or in mouse (The Developmental Studies Hybridoma Bank) were used at a dilution of 1∶5000 and 1∶50 respectively. Anti-Twist antibody (Siegfried Roth, University of Köln), was used pre-adsorbed at a dilution of 1∶500 to late stage (15–16) embryos and then used. Anti-Tinman antibody (Manfred Frasch) was used at a dilution at 1∶200, Anti-Evenskipped (Nipam Patel) at 1∶10, Anti Kr at 1∶500, Mouse 22C10 (The Developmental Studies Hybridoma Bank) at 1∶75 and Anti GFP (Molecular Probes) at 1∶500. Secondary antibodies conjugated to Alexa Fluor dyes- Alexa 488 and Alexa 568 (from Molecular Probes, Eugene, OR) was used. Fluorescent preparations were scanned using the confocal microscopes (MRC-1024, BioRad Laboratories, Hercules, CA) or Ziess LSM 510Meta (Carl Zeiss GmbH). Raw images were analyzed using Confocal Assistant (version 4.02) or Ziess Image Viewer (version 3,2,0,70. Carl Zeiss GmbH) and processed in Adobe® Photoshop® CS3 version 10.0.

### Bioinformatics

For initial screen of *duf* genomic region for potential TF binding sites, Matinspector Professional® [38] was used. Additional binding sites information for nuclear effectors of important signaling pathways and some mesoderm specific factors from original published work were also used. The consensus sequences for a combination of signaling pathways such as E-twenty six (Ets) [NSYGGAWRY] [13] downstream of RTK pathway, Mothers against Dpp (Mad) [GCCGNCGC] [59] an activator of Dpp pathway, Cubitus interruptus (Ci) [TGGGWGGTC] [60] of Hh pathway, and Brinker (Brk) [TGGCGYY] [61], [62] a transcriptional repressor of Dpp and Wg pathways were used. All these factors, except Brk, have been shown to have important roles in early mesodermal development, cell fate specification, founder selection and muscle differentiation [13], [49]. Similarly, consensus binding sites for important mesodermal factors such as Twi [CACATGT] [13], Tin [TYAAGTG] [63] and [TCAAGTGG] [64], Mef2 [YTAWWWWTAR] [65], CF2 [RTATATRTA] [66], [67] and PDP1 [RTTTWAYGTAAY] [68] were integrated into our search for *cis-*regulatory elements regulating *duf* expression in FCs.

### Position Weight matrices

44 weight matrices for transcription factors, or groups of related homeodomain factors, were chosen based on their known relevance to mesoderm development (as given by their flybase annotations or literature references) and availability of position weight matrices or binding site data. For the factors bin, brk, byn, dl, en, E(spl), Mad, pan, sna, Su(H), twi, br-Z1, br-Z2, br-Z3, br-Z4, Cf2-II, Eip74EF, exd, ey, gsb, gsb-n, Med, prd, srp, toy, sd, Trl, matrices were constructed from DNAse-I footprints available for *D. melanogaster* in the Flyreg database [32], and orthologous sequence from *D. simulans, D. yakuba, D. erecta, D. ananassae, D. pseudoobscura*, using PhyloGibbs-MP as described in [31]. For Kr, kni and ttk, matrices were constructed from binding sequences reported in bacteria-one-hybrid (B1H) experiments from [43]. For eight groups of homeodomain factors, namely Abd-B group (Abd-B, Cad), Antp group (Antp, AbdA, Ubx, Dfd, Zen), Ap group (Ap), Bcd group (Bcd, Ct), En group (En, Dr), Ladybird group (Eve, Ftz), NK1 group (Bsh, Slou, Dll), and NK2 group (Tin, Bap, Vnd), matrices (one matrix per group) were constructed from B1H data in [33]. Raw sequences for multiple factors (as listed above) were aligned for each group, since their binding motifs are extremely similar and probably not distinguishable via our bioinformatic tools. Matrices from other sources were STAT [69] (from the Transfac 7 database [70]), shn1 and shn2 (the two binding domains of shn, from Dai et al. [71]), Ci (constructed from the consensus sequence TGGGTGGTC, with consensus bases given a weight of 0.85 and the remainder distributed uniformly), Mef2 (from binding sequences from Elgar et al. [72]) and Pnt (from Halfon et al. [73]). Sequence logos for all these weight matrices are in Supplementary data Figure S5. Only the first 31 matrices listed there were actually used. The raw weight matrix files are included in Supplementary Datasets S1, S2, S3.

### Enhancer prediction: Stubb

Stubb [19], [20] version 2.1 was run on the 30 kb region upstream of *duf*, and in the approximately 29 kb intron, using these 31 input weight matrices. In release 4 coordinates, the upstream region selected was 2926233–2956233 and the intron was 2956475–2985408, on chromosome X. Orthologous sequence from *D. pseudoobscura* was selected (using the alignments cited above), and Stubb was run on these sequences in multi-sequence mode. (As part of the process of running Stubb, these sequences were re-aligned with LAGAN.) The raw input and output files and exact command lines are in Supplementary Dataset S1.

### Enhancer prediction: PhyloGibbs-MP

PhyloGibbs-MP was run, with these prior weight matrices and in “module-finding” mode, on the same upstream and intronic regions. The upstream region used was 2926000–2956000, and the intron region 2956475–2985408, on chromosome X. Orthologous sequence was taken from *D. pseudoobscura, D. yakuba, D. simulans* (again, from the alignments of Eisen et al. cited above), and re-aligned with Sigma. (We used only four species, including melanogaster, because including more species slows down the performance of PhyloGibbs-MP without noticeably improving results.) In this run, PhyloGibbs-MP searched for motifs in 500bp contiguous regions, separated on average by 500 bp (thus expecting that about half each region is occupied by cis-regulatory modules), and within each such region, on each sequence, the probability of a given site being a binding site (for any factor) is 0.005. Upto 55 motifs are sought (of which up to 31 may be associated with the prior PWMs), and each 500 bp module may contain up to 40 motifs. The raw input and output files and exact command lines are in Supplementary Dataset S2.

### Analysis of 11 kb upstream region: PhyloGibbs-MP

The region 2945000–2956000 (about 11 kb upstream of *duf*) was examined in detail. Sequence preparation was as described above, but PhyloGibbs-MP was run treating all of the sequence as potentially regulatory (the specified “module dimension” being the entire length of the sequence). Also, after preliminary runs revealed poor or non-specific predictions for many factors, the list of input factors was trimmed to the following: bin, byn, E(spl), toy, kni, shn1, shn2, STAT, Ci, dmef, brk, dl, Mad, pan, Su(H), twi, Cf2-II, Eip74EF, exd, Kr, Med, prd, sd, Trl, Pnt, Abd-B group, Antp group, Ap group, Bcd group, Ladybird group, NK2 group. These were separated into two groups, one with consensus motifs 8 bp long or shorter, and the other with longer consensus motifs, because PhyloGibbs-MP cannot handle differing lengths of motifs and, while robust to small variations, will show inferior performance with highly varying motif sizes. The raw input and output files and exact command lines are in Supplementary Dataset S3.

## Supporting Information

Datasheet S1 MatInspector results for potential TF binding sites in 10 kb *duf* upstream region. This table lists MatInspector results for potential binding sites for different TFs in 10 kb *duf* upstream region. Also listed are occurrences of potential TF binding sites that match the published consensus binding sites for signalling and mesodermal factors (shown in bold). The arrangement of these putative binding sites found in *duf* upstream region along with primer binding sites used for cloning different *duf* enhancer constructs are shown. Putative GAGA binding sites characteristic of TREs and PHO and PHO-like binding sites PREs are also highlighted.
(0.07 MB XLS)

Datasheet S2 Conserved TF binding sites in the −10 kb *duf* upstream sequence between *Drosophila melanogaster* and *Drosophila pseudoobscura*. This table lists putative binding sites in the 10 kb upstream of *duf* from *Drosophila melanogaster* compared with that of *Drosophila pseudoobscura*. The + or − sign in the parenthesis indicating the location of the binding site on the coding or non-coding strand in this sequence respectively. Primer binding sites used for cloning are shown in bold green. Light blue and pale green numbers indicate the stretch of sequence identical between the two genomes. Sites highlighted in red (signaling pathways) and green (transcription factors) are well conserved. Those in orange are weakly conserved; those in light yellow are conserved in position/location and those shown in white are unique to *D. melanogaster*.
(0.11 MB XLS)

Datasheet S3 Predictions from Stubb and PhyloGibbs-MP that match several published consensus sites. This table shows comparison of different predictions of putative TF binding sites using Stubb, PhyloGibbs-MP, and MatInspector Professional® and published consensus sequences in −10 kb *duf* enhancer region. Matching predictions are highlighted along with the sequence and distance from *duf* start site and primers used for cloning the enhancer fragments.
(0.05 MB XLS)

Figure S1 Additional *duf* enhancer reporter deletion constructs. Additional set of enhancer deletion constructs available for studying transcriptional regulation of *duf*. Preliminary analysis has been carried out on some of the constructs. *duf −0.6 to 6.0 kb* and *duf 6.0 to 8.5 kb* (magenta bars) covering part of the first intron sequence were tested and they are not expressed in any muscles. *duf −14 to −15 kb Gal4* showed no expression in mesoderm/muscles. This small fragment has ectopic expression in epidermal cells that appears to be apodemes (muscle attachment sites within the epidermis). Others constructs are available as sequence verified plasmids for further detailed analysis of *duf* enhancer region.
(0.20 MB TIF)

Figure S2 Expression pattern of additional *duf* enhancer reporter deletion constructs. Lateral view confocal images of embryos from different *duf* enhancer reporter lines showing reporter expression during important embryonic myogenesis. Reporter expression was assayed by using antibodies against βGal. Gal4 transgenic lines were crossed with *UAS-lacZ*. A. Stage 16 *duf Gal4* embryo showing expression in all the somatic (body wall) muscles. In comparison, *duf −3.8 kb Gal4* reporter expression (B) is seen not in all, but a large subset of somatic muscles. *duf −3.8 kb Gal4* shows clear expression in easily identifiable subset of somatic muscle FCs at stage 13 (shown in C). In comparison, in lacZ version of the same construct (*duf −3.8 kb lacZ* shown in D), reporter expression appears diffuse in some somatic FCs. Whereas, in a slightly larger construct *duf −4.6 kb lacZ* (shown in C), the reporter expression is more stronger in majority of the somatic muscle FCs. *duf −7.9−3.8 kb Gal4* reporter expression is specifically seen in circular visceral muscle FCs (arrow in F and G) and few somatic FCs (white arrow heads G and H). Ectopic expression is also seen in some other cells at later stages (red arrow heads in G and H). *duf −7.2 kb lacZ* is expressed in all the somatic muscle FCs (arrow heads in I and J) and also in circular visceral muscles (arrows in J) revealed by optical slices closer to the centre of the embryo. Reporter expression of *duf −7.2 kb lacZ* is weak compared to other reporter constructs. *duf −6.4 kb lacZ* (K-M) shows ectopic expression in the epidermis and trachea (red arrow heads in L and M) and very weak expression in the somatic muscles. *duf −4.6−3.0 kb Gal4* (N and O) shows ectopic reporter expression the epidermis. Scale bar  = 50 microns.
(1.90 MB TIF)

Figure S3 *duf* enhancer lacZ reporter expression colocalizes with *duf Gal4; UAS GFP* in stage 14 embryos. Confocal projections of different *duf* enhancer lacZ reporter construct with *duf Gal4; UAS GFP* in stage 14 embryos. The embryos are double labeled with antibodies against β galactosidase (in red) to show reporter expression and GFP (in green) to report wildtype *duf* expression in muscle FCs driven by *duf Gal4*. The size of the construct is indicated on panel showing colocalization (in yellow). Expression of different *duf* enhancer lacZ constructs is seen in specific founders that give rise to different muscles of the embryo. Expression in the visceral muscles is obscured by the overlying somatic muscles. Complete colocalization with *duf Gal4* driven GFP is seen in somatic muscles of *duf −5.3 kb lacZ* embryos. Lateral view embryos with anterior is to the left and dorsal to the top. Scale Bar = 100 microns.
(2.53 MB TIF)

Figure S4 Descripton of ectopic expression observed in *duf −2.4 kb lacZ* and *duf −3.0 kb lacZ*. Confocal projections of *duf* enhancer lacZ constructs showing ectopic reporter expression in cardioblasts and neuroblasts. Top panels are stage 16 dorsal view of *duf Gal4; UAS lacZ* and *duf −2.4 kb lacZ* embryos double labeled with antibodies against β galactosidase (green) and Tin (magenta) to mark the cardioblasts that form the heart. Bottom panels are stage 16 ventral view of *duf −3.0 kb lacZ* and *rP298 (duf) lacZ* embryos double labeled with antibodies against β galactosidase (green) and Eve (red) to mark large subset of neuroblasts. Box region is ∼125 microns covering A2-A5 abdominal segments in the top panel, and A2-A6 in the bottom panel, magnified to show details in individual channels. *duf −2.4 kb lacZ* is strongly expressed in two cells that do not express Tinman and weakly in other in cardioblasts (arrows in D – F). Wildtype *duf* expression in *duf Gal4; UAS lacZ* is not detected in developing cardioblasts (arrow in B). Wildtype *duf* is expressed in CNS midline by stage 16 (small arrows in H). *duf −3.0 kb lacZ* is also expressed in the CNS but in a larger subset of neuroblasts (K) when compared to wildtype *duf* (H). (All images- anterior to the left).
(3.69 MB TIF)

Figure S5 Sequence logos corresponding to position weight matrices generated by PhyloGibbs-MP. Sequence logos corresponding to position weight matrices generated by PhyloGibbs-MP from FlyReg DNAse I footprints database [32] or recent data from bacteria-one-hybrid systems [33]. In addition, logos corresponding to five factors from the literature are included. A subset of 31 PWMs was eventually used, the remainder (13 PWMs) being poor-quality (not specific enough) or not making significant predictions in preliminary runs. The sequence logos were made with Weblogo 2.8 [75]. These PWMs were used as prior information for the PhyloGibbs-MP and Stubb runs reported in Figures 5 and 6.
(0.18 MB PDF)

Figure S6 Additional PhyloGibbs-MP and Stubb Results for 11 kb *duf* Upstream. Predictions of individual binding sites for various factors from Stubb and PhyloGibbs-MP in the *duf* 11 kb region. For both programs, weight matrices for 31 mesoderm-relevant factors, as discussed in the text, were used as priors. The first set of high priority factors are shown and discussed in Fig. 6. The second set of homeo domain factors with some relevance to mesoderm development are shown here. PWMs for several homeodomain factors have very similar core structures so there is significant competition between predictions for these factors. The sequence logos were made with Weblogo 2.8 [75]. The predictions were plotted with our genome visualization tool (S. Acharya and R. Siddharthan, unpublished).
(0.82 MB TIF)

Table S1 Details of the primers designed to amplify different *duf* upstream genomic fragments. Specific primers were designed to PCR different upstream fragments of *duf*. Most of the forward primers (F) have *EcoRI* (E) and reverse primers (R) have either *BamHI* (B) or *EcoRI* as the ectopic restriction site, which were included in the 5′ end of the primers as overhangs to assist in cloning. An additional five to eight bases were added after the restriction site to the primer for improved digestion by the restriction enzymes. The primer matches the target sequence exactly 3′ to the restriction site (shown as a slash). Distance (in base pairs) from *duf* transcription start site is indicated as start and end.
(0.04 MB DOC)

Dataset S1 The raw input and output files of enhancer predictions using Stubb. Stubb version 2.1 was run on the 30 kb region upstream of *duf* and approximately 29 kb intron, using 45 input weight matrices. In release 4 coordinates, the upstream region selected was 2926233–2956233 and the intron was 2956475–2985408, on chromosome X. Orthologous sequence from *D. pseudoobscura* was selected. As part of the process of running Stubb, these sequences were re-aligned with LAGAN and Stubb was run on these sequences in multi-sequence mode. The raw input and output files are in Supplementary Data S11.
(4.59 MB ZIP)

Dataset S2 The raw input and output files of enhancer predictions using PhyloGibbs-MP. PhyloGibbs-MP was run, with these prior weight matrices and in “module-finding” mode, on the same upstream and intronic regions. The upstream region used was 2926000–2956000, and the intron region 2956475–2985408, on chromosome X. Orthologous sequence was taken from *D. pseudoobscura, D. yakuba, D. simulans* and re-aligned with Sigma. The raw output files are in Supplementary Data S12.
(0.12 MB ZIP)

Dataset S3 The raw output of the analysis of 11 kb upstream region using PhyloGibbs-MP. The region 2945000–2956000 (about 11 kb upstream of *duf*) was examined in detail. Sequence preparation was as described above, but PhyloGibbs-MP was run treating 2/3 of the sequence, rather than half the sequence, as potentially regulatory (the specified “module dimension” being the entire length of the sequence). The raw output is in Supplementary data 13.
(0.06 MB ZIP)
