# Supplementary figures and images for: The Complex Spatio-Temporal Regulation of the Drosophila Myoblast Attractant Gene duf/kirre
Source: PLoS One. 2009 Sep 9;4(9):e6960. doi: 10.1371/journal.pone.0006960 (PMC2734059; doi:10.1371/journal.pone.0006960)

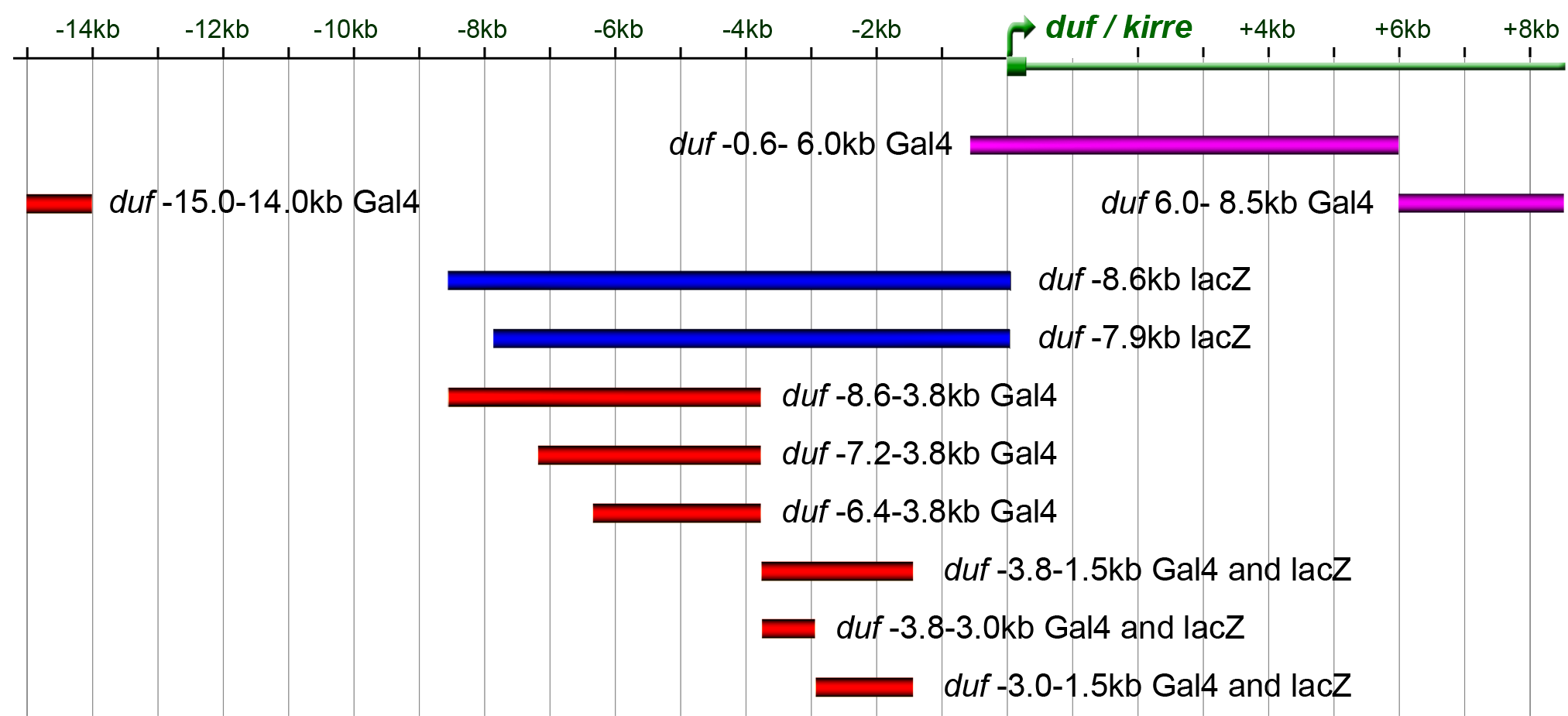

Supplement: Figure S1 — Additional duf enhancer reporter deletion constructs. Additional set of enhancer deletion constructs available for studying transcriptional regulation of duf. Preliminary analysis has been carried out on some of the constructs. duf −0.6 to 6.0 kb and duf 6.0 to 8.5 kb (magenta bars) covering part of the first intron sequence were tested and they are not expressed in any muscles. duf −14 to −15 kb Gal4 showed no expression in mesoderm/muscles. This small fragment has ectopic expression in epidermal cells that appears to be apodemes (muscle attachment sites within the epidermis). Others constructs are available as sequence verified plasmids for further detailed analysis of duf enhancer region. (0.20 MB TIF) [file pone.0006960.s004.tif]

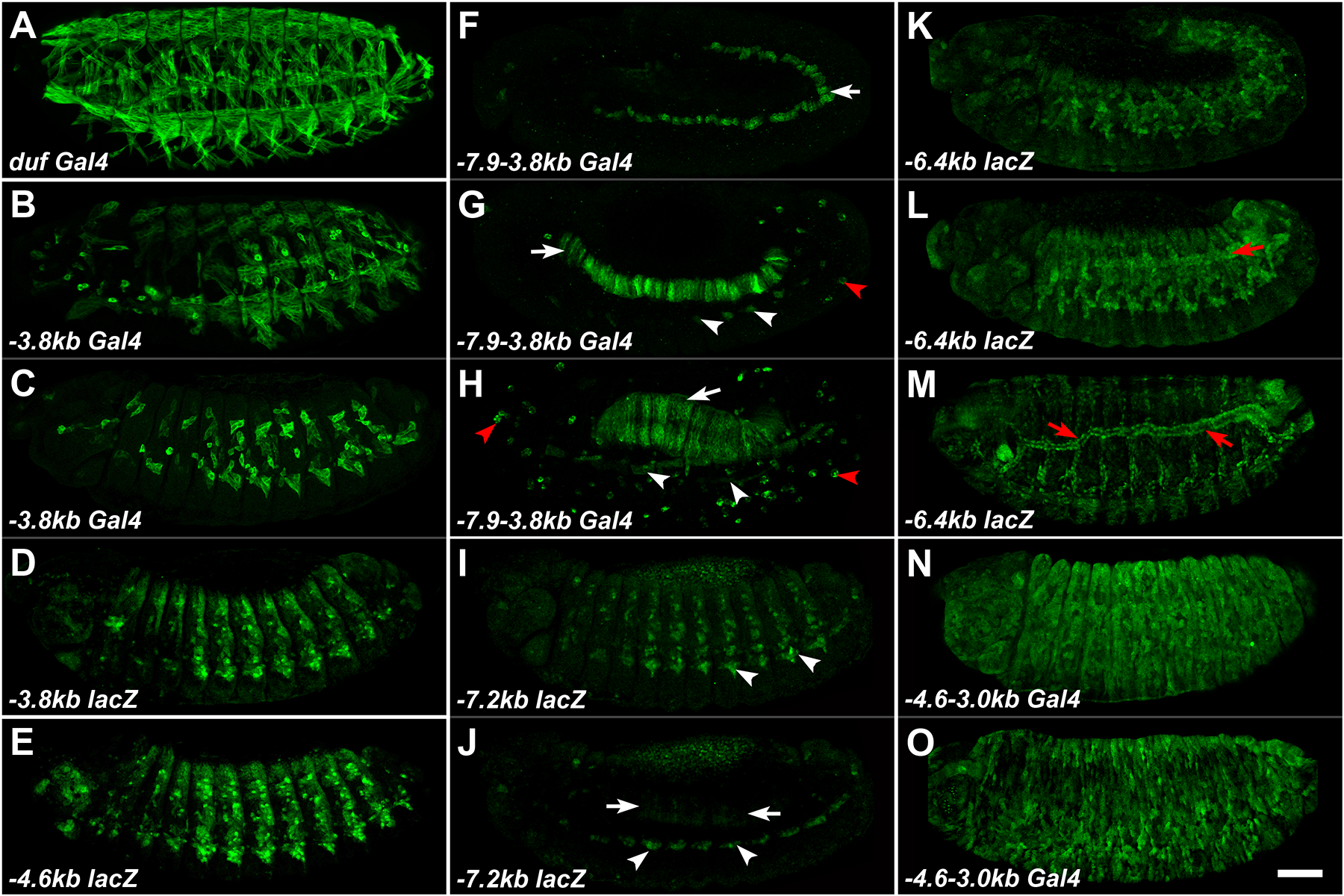

Supplement: Figure S2 — Expression pattern of additional duf enhancer reporter deletion constructs. Lateral view confocal images of embryos from different duf enhancer reporter lines showing reporter expression during important embryonic myogenesis. Reporter expression was assayed by using antibodies against βGal. Gal4 transgenic lines were crossed with UAS-lacZ. A. Stage 16 duf Gal4 embryo showing expression in all the somatic (body wall) muscles. In comparison, duf −3.8 kb Gal4 reporter expression (B) is seen not in all, but a large subset of somatic muscles. duf −3.8 kb Gal4 shows clear expression in easily identifiable subset of somatic muscle FCs at stage 13 (shown in C). In comparison, in lacZ version of the same construct (duf −3.8 kb lacZ shown in D), reporter expression appears diffuse in some somatic FCs. Whereas, in a slightly larger construct duf −4.6 kb lacZ (shown in C), the reporter expression is more stronger in majority of the somatic muscle FCs. duf −7.9−3.8 kb Gal4 reporter expression is specifically seen in circular visceral muscle FCs (arrow in F and G) and few somatic FCs (white arrow heads G and H). Ectopic expression is also seen in some other cells at later stages (red arrow heads in G and H). duf −7.2 kb lacZ is expressed in all the somatic muscle FCs (arrow heads in I and J) and also in circular visceral muscles (arrows in J) revealed by optical slices closer to the centre of the embryo. Reporter expression of duf −7.2 kb lacZ is weak compared to other reporter constructs. duf −6.4 kb lacZ (K-M) shows ectopic expression in the epidermis and trachea (red arrow heads in L and M) and very weak expression in the somatic muscles. duf −4.6−3.0 kb Gal4 (N and O) shows ectopic reporter expression the epidermis. Scale bar = 50 microns. (1.90 MB TIF) [file pone.0006960.s005.tif]

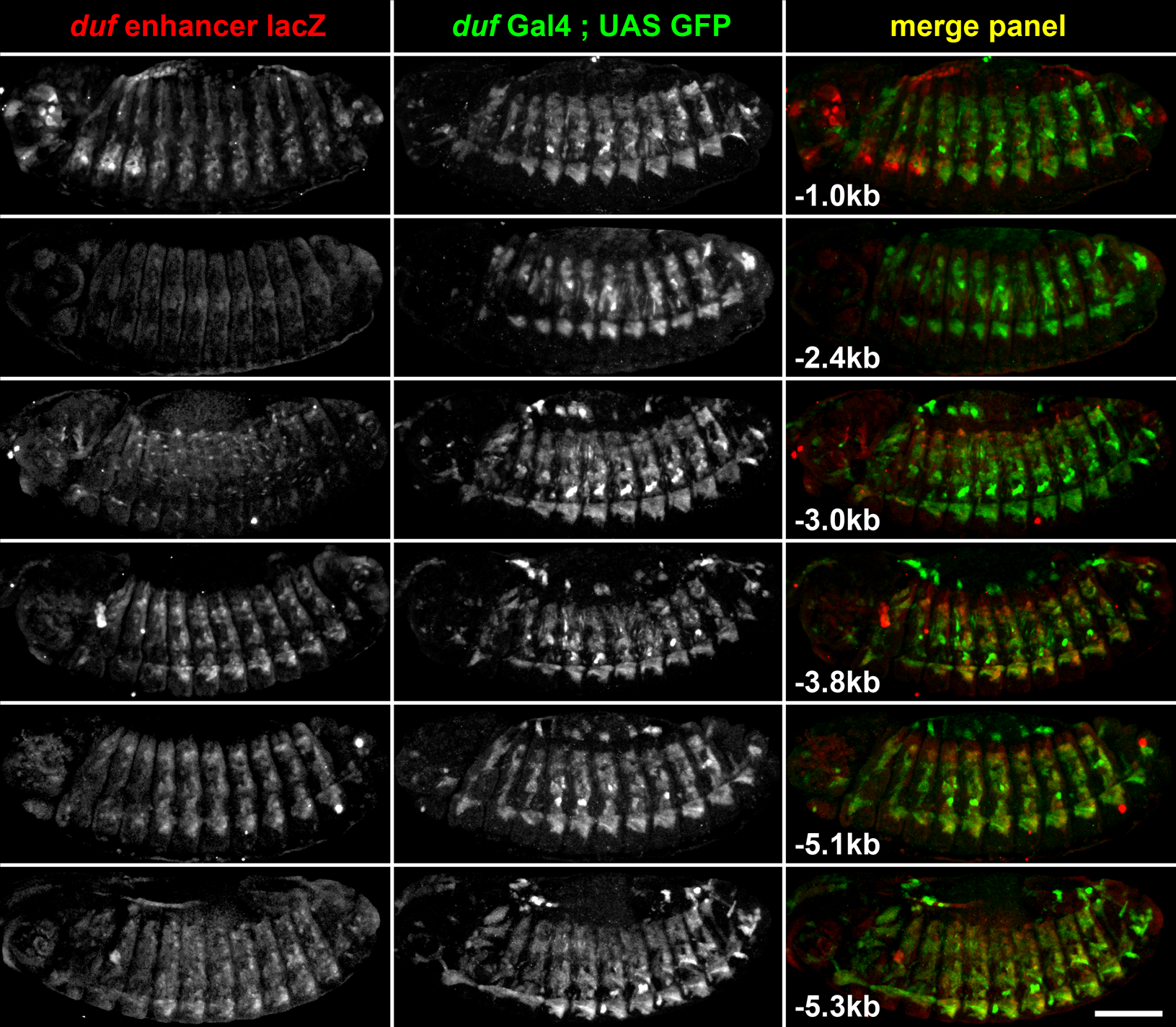

Supplement: Figure S3 — duf enhancer lacZ reporter expression colocalizes with duf Gal4; UAS GFP in stage 14 embryos. Confocal projections of different duf enhancer lacZ reporter construct with duf Gal4; UAS GFP in stage 14 embryos. The embryos are double labeled with antibodies against β galactosidase (in red) to show reporter expression and GFP (in green) to report wildtype duf expression in muscle FCs driven by duf Gal4. The size of the construct is indicated on panel showing colocalization (in yellow). Expression of different duf enhancer lacZ constructs is seen in specific founders that give rise to different muscles of the embryo. Expression in the visceral muscles is obscured by the overlying somatic muscles. Complete colocalization with duf Gal4 driven GFP is seen in somatic muscles of duf −5.3 kb lacZ embryos. Lateral view embryos with anterior is to the left and dorsal to the top. Scale Bar = 100 microns. (2.53 MB TIF) [file pone.0006960.s006.tif]

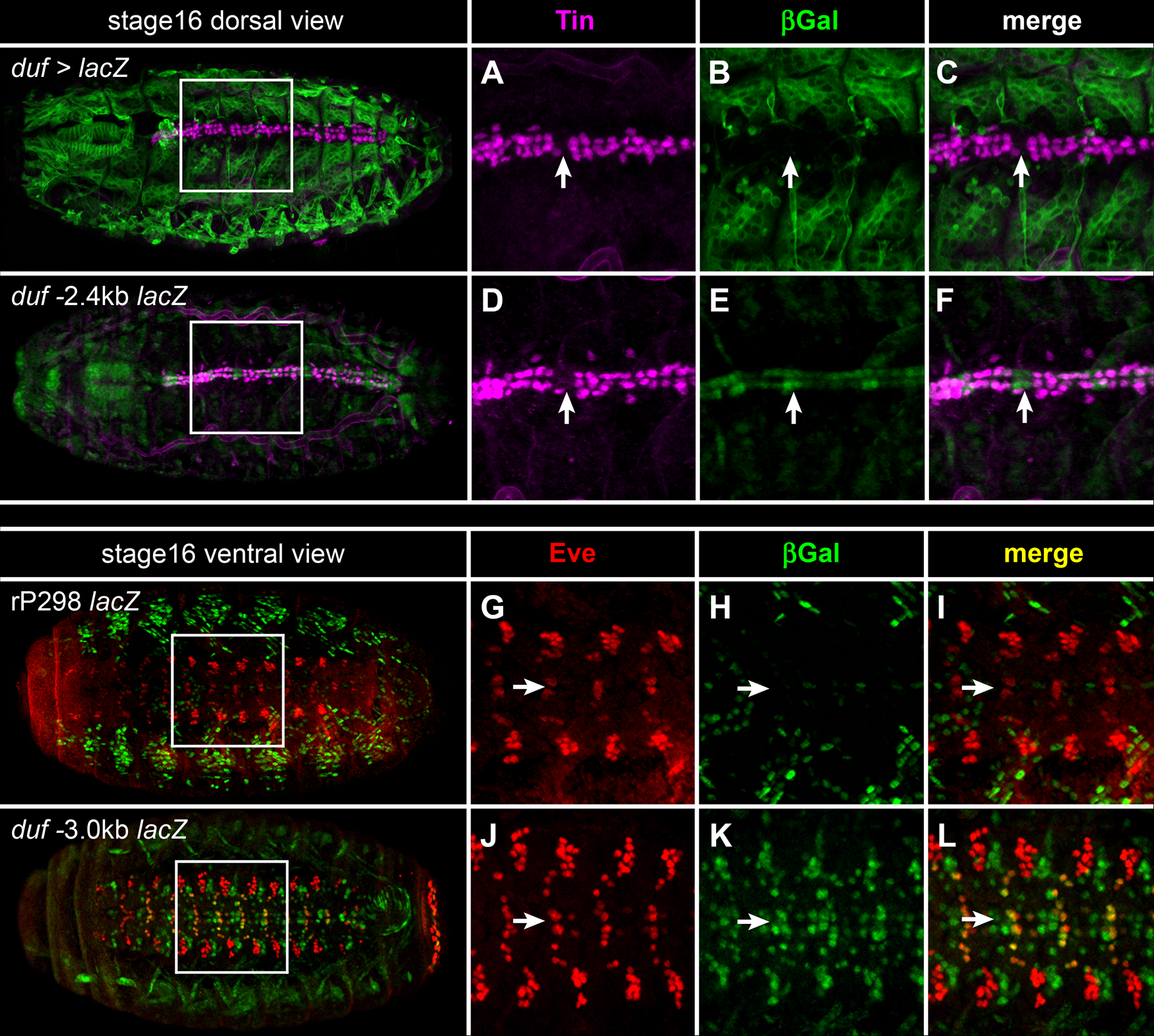

Supplement: Figure S4 — Descripton of ectopic expression observed in duf −2.4 kb lacZ and duf −3.0 kb lacZ. Confocal projections of duf enhancer lacZ constructs showing ectopic reporter expression in cardioblasts and neuroblasts. Top panels are stage 16 dorsal view of duf Gal4; UAS lacZ and duf −2.4 kb lacZ embryos double labeled with antibodies against β galactosidase (green) and Tin (magenta) to mark the cardioblasts that form the heart. Bottom panels are stage 16 ventral view of duf −3.0 kb lacZ and rP298 (duf) lacZ embryos double labeled with antibodies against β galactosidase (green) and Eve (red) to mark large subset of neuroblasts. Box region is ∼125 microns covering A2-A5 abdominal segments in the top panel, and A2-A6 in the bottom panel, magnified to show details in individual channels. duf −2.4 kb lacZ is strongly expressed in two cells that do not express Tinman and weakly in other in cardioblasts (arrows in D – F). Wildtype duf expression in duf Gal4; UAS lacZ is not detected in developing cardioblasts (arrow in B). Wildtype duf is expressed in CNS midline by stage 16 (small arrows in H). duf −3.0 kb lacZ is also expressed in the CNS but in a larger subset of neuroblasts (K) when compared to wildtype duf (H). (All images- anterior to the left). (3.69 MB TIF) [file pone.0006960.s007.tif]

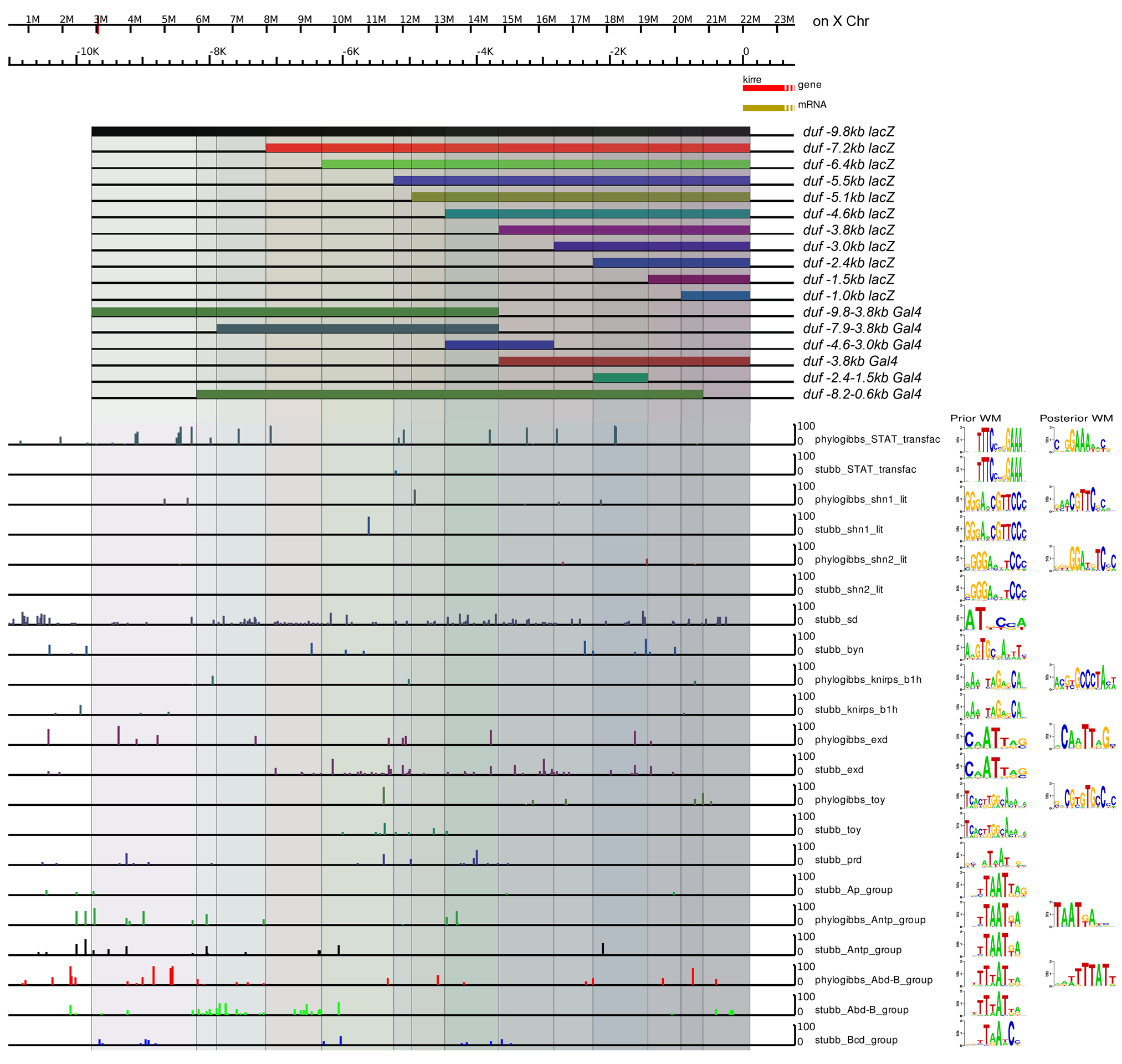

Supplement: Figure S6 — Additional PhyloGibbs-MP and Stubb Results for 11 kb duf Upstream. Predictions of individual binding sites for various factors from Stubb and PhyloGibbs-MP in the duf 11 kb region. For both programs, weight matrices for 31 mesoderm-relevant factors, as discussed in the text, were used as priors. The first set of high priority factors are shown and discussed in Fig. 6. The second set of homeo domain factors with some relevance to mesoderm development are shown here. PWMs for several homeodomain factors have very similar core structures so there is significant competition between predictions for these factors. The sequence logos were made with Weblogo 2.8 [75]. The predictions were plotted with our genome visualization tool (S. Acharya and R. Siddharthan, unpublished). (0.82 MB TIF) [file pone.0006960.s009.tif]
