# Supplementary material for: The Complex Spatio-Temporal Regulation of the Drosophila Myoblast Attractant Gene duf/kirre
Source: PLoS One. 2009 Sep 9;4(9):e6960. doi: 10.1371/journal.pone.0006960 (PMC2734059; doi:10.1371/journal.pone.0006960)

The complex spatio-temporal regulation of the Drosophila myoblast attractant gene duf/ kirre

(K G Guruharsha et al.)

Supporting data S5: Sequence logos for factors used as priors by Stubb and PhyloGibbs-MP

(For full references / details of construction, see main paper)

---

bin (from Flyreg)

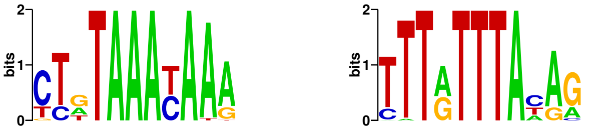

byn (from Flyreg)

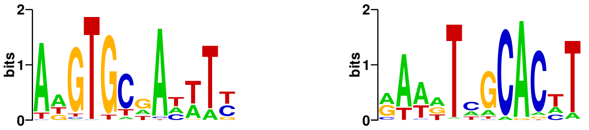

E(spl) (from Flyreg)

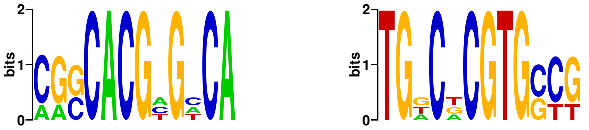

toy (from Flyreg)

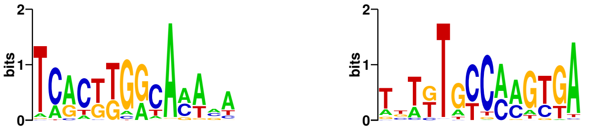

brk (from Flyreg)

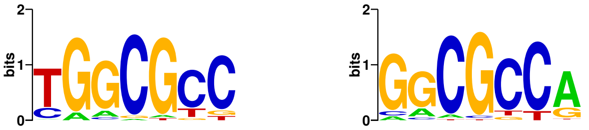

dl (from Flyreg)

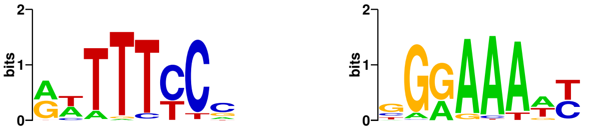

Mad (from Flyreg)

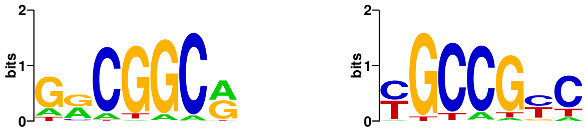

pan (from Flyreg)

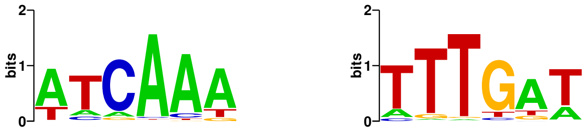

Su(H) (from Flyreg)

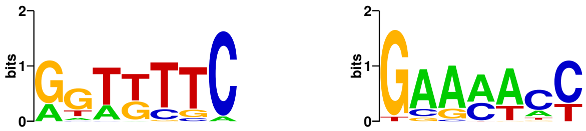

twi (from Flyreg)

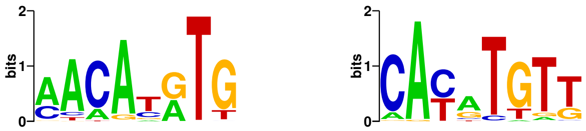

Cf2-II (from Flyreg)

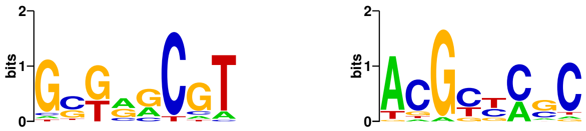

Eip74EF (from Flyreg)

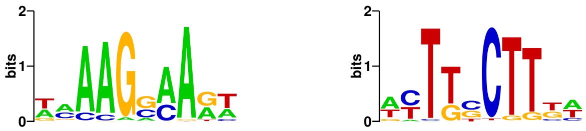

exd (from Flyreg)

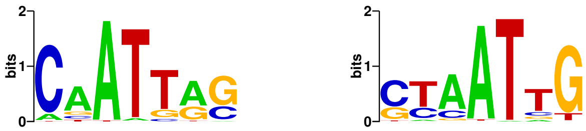

Med (from Flyreg)

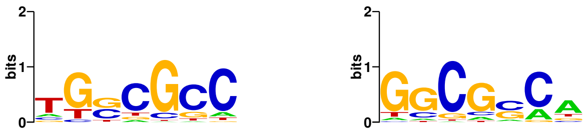

prd (from Flyreg)

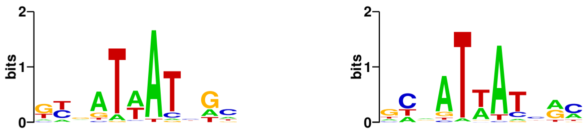

sd (from Flyreg)

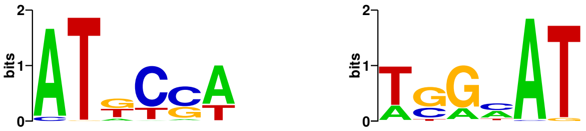

Trl (from Flyreg)

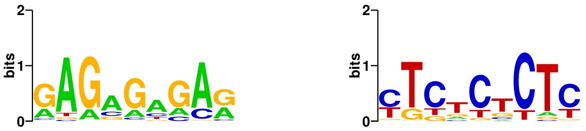

knirps (from B1H data, Noyes et al)

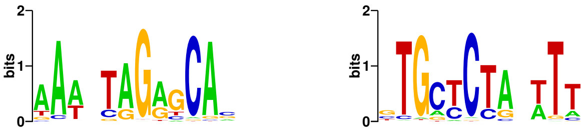

kruppel (from B1H data, Noyes et al)

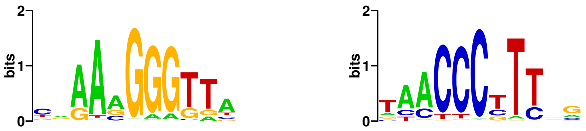

Abd-B homeodomain group (from B1H data, Noyes et al)

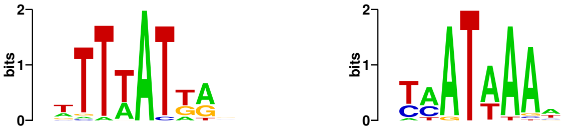

Antp homeodomain group (from B1H data, Noyes et al)

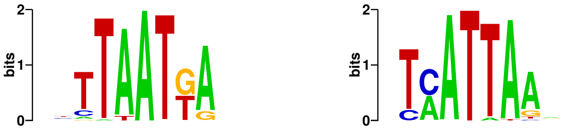

Ap homeodomain group (from B1H data, Noyes et al)

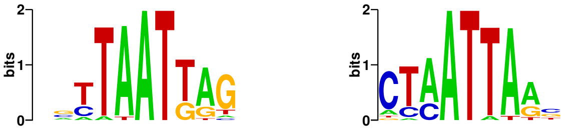

Bcd homeodomain group (from B1H data, Noyes et al)

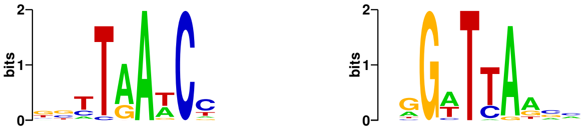

Ladybird homeodomain group (from B1H data, Noyes et al)

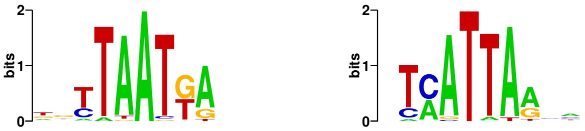

NK2 homeodomain group (from B1H data, Noyes et al)

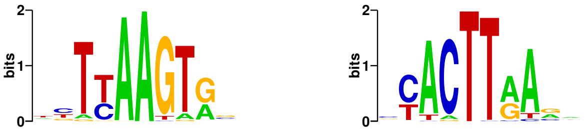

STAT (from Transfac database)

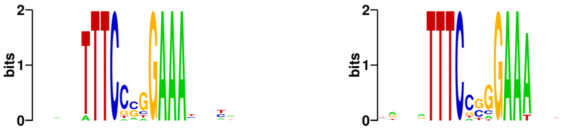

shn1 (from literature)

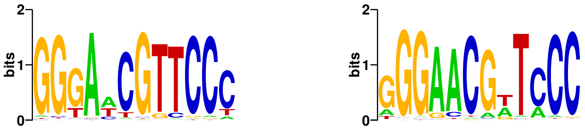

shn2 (from literature)

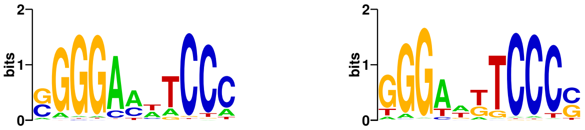

dmef (from literature)

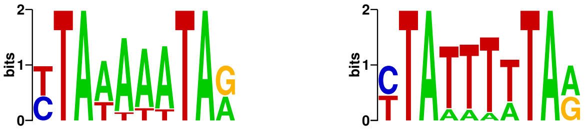

Pnt (from literature)

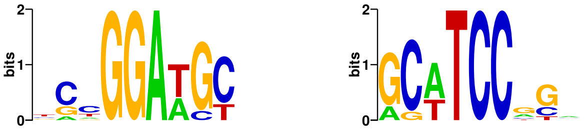

Ci (from consensus sequence)

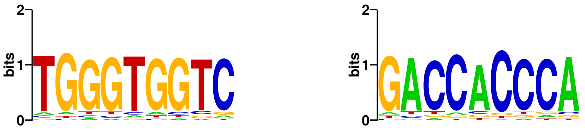

(The following factors were considered but rejected, either because the available weight matrices were not specific enough, or because preliminary runs did not show significant predictions)

br-Z1 (from Flyreg)

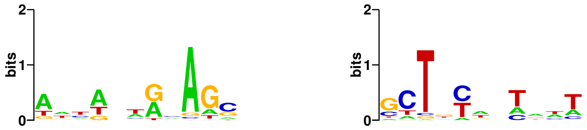

br-Z2 (from Flyreg)

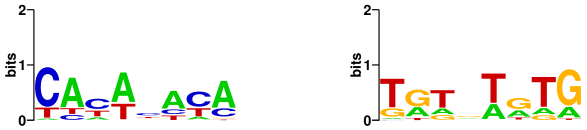

br-Z3 (from Flyreg)

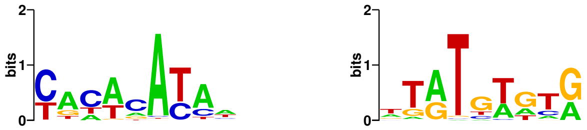

br-Z4 (from Flyreg)

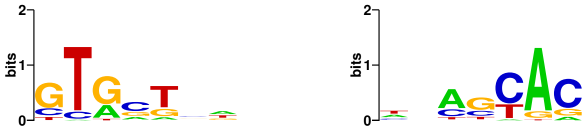

ey (from Flyreg)

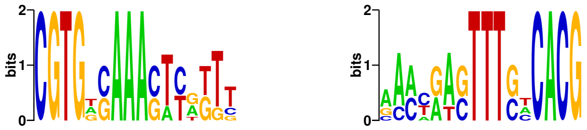

gsb-n (from Flyreg)

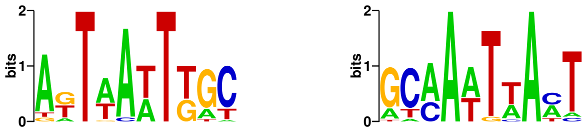

gsb (from Flyreg)

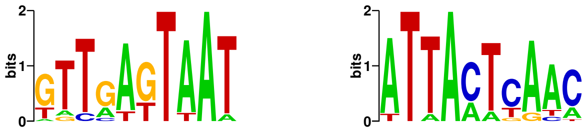

sna (from Flyreg)

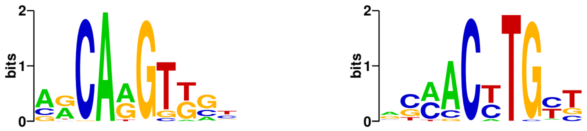

srp (from Flyreg)

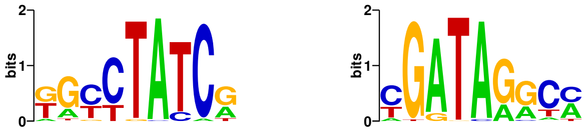

vnd (from Flyreg)

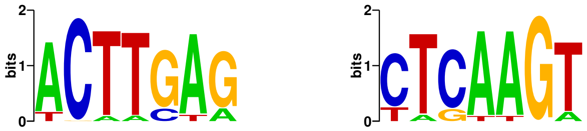

ttk (from B1H data, Noyes et al)

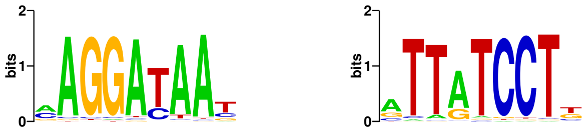

En homeodomain group (from B1H data, Noyes et al)

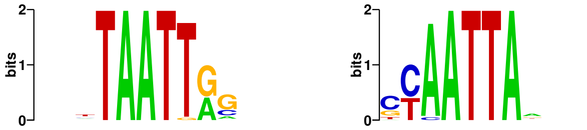

NK1 homeodomain group (from B1H data, Noyes et al)

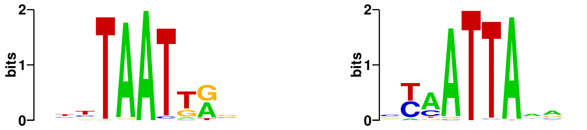

Supplement: Figure S5 — Sequence logos corresponding to position weight matrices generated by PhyloGibbs-MP. Sequence logos corresponding to position weight matrices generated by PhyloGibbs-MP from FlyReg DNAse I footprints database [32] or recent data from bacteria-one-hybrid systems [33]. In addition, logos corresponding to five factors from the literature are included. A subset of 31 PWMs was eventually used, the remainder (13 PWMs) being poor-quality (not specific enough) or not making significant predictions in preliminary runs. The sequence logos were made with Weblogo 2.8 [75]. These PWMs were used as prior information for the PhyloGibbs-MP and Stubb runs reported in Figures 5 and 6. (0.18 MB PDF) [file pone.0006960.s008.pdf]
