# Supplementary material for: The Complex Spatio-Temporal Regulation of the Drosophila Myoblast Attractant Gene duf/kirre
Source: PLoS One. 2009 Sep 9;4(9):e6960. doi: 10.1371/journal.pone.0006960 (PMC2734059; doi:10.1371/journal.pone.0006960)
Supplement: Table S1 — Details of the primers designed to amplify different duf upstream genomic fragments. Specific primers were designed to PCR different upstream fragments of duf. Most of the forward primers (F) have EcoRI (E) and reverse primers (R) have either BamHI (B) or EcoRI as the ectopic restriction site, which were included in the 5′ end of the primers as overhangs to assist in cloning. An additional five to eight bases were added after the restriction site to the primer for improved digestion by the restriction enzymes. The primer matches the target sequence exactly 3′ to the restriction site (shown as a slash). Distance (in base pairs) from duf transcription start site is indicated as start and end. (0.04 MB DOC) [file pone.0006960.s010.doc]

**Table S10. Details of the primers designed to amplify different *duf* upstream genomic fragments.**

| start | end | Name | Sequence |
| --- | --- | --- | --- |
| 89 | 112 | Duf RBx | gtataagcg/gatccaagtgtgatatcgtgtttggcc |
| -935 | -912 | Duf 1 FEx | gtttagaccg/aattccttaacatagaagccgacagcc |
| -1407 | -1428 | Duf 1.5 RBx | gtatagtacg/gatcctcgactgtaccaccttaggtgg |
| -1428 | -1407 | Duf 1.5 FEx | gttcaacacg/aattccacctaggtgtacagtcgag |
| -2254 | -2230 | Duf 2.4 FEx | gctgcatggcg/aattcaaaccgaattataagtggagtg |
| -2839 | -2815 | Duf 3.0 FEx | gtccatgacg/aattctatgtcctgtctcgcatttcg |
| -3639 | -3664 | Duf 3.8 REx | gtatcacacg/aattccgcataatcgagggcggaac |
| -3645 | -3668 | Duf 3.8 RBx | gttaatacacg/gatccataatcgagggcggaacaacgg |
| -3668 | -3645 | Duf 3.8 FEx | gttacatacg/aattccgttgttccgccctcgattatg |
| -4477 | -4450 | Duf 4.6 FEx | gtccaaccgg/aattctcgttagactcgtaattatattgcc |
| -4967 | -4946 | Duf 5.1 FEx | gtataaccgg/aattcccttccacattggtcctctc |
| -5236 | -5211 | Duf 5.3 FEx | cctacgctag/atttcactatgcctgactagtttctacg |
| -5414 | -5390 | Duf 5.5 FEx | cctaagctag/aattcgagcgcaattattgacgtgtgc |
| -6321 | -6294 | Duf 6.4 FEx | caatggatgtg/aattcgccatggggagtttaagccaacacgc |
| -6438 | -6413 | Duf 6.5 FEx | cctgaggattg/aattcactggacggacgggctagatac |
| -7158 | -7134 | Duf 7.2 FEx | ctcgagaagcg/aattcgctgacgcagcgggtttttgggc |
| -7851 | -7825 | Duf 7.9 FEx | cgagtcgattg/aattcatgctgttggttggtgcgagggagag |
| -8557 | -8536 | Duf 8.6 FEx | cgaattcgatg/aattcaagcgcgcaccaagctggcgaag |
| -9474 | -9437 | Duf 9.5 FEx | gccaaactgttgg/aattccgctcatcttgcctttccggtc |
| -9721 | -9740 | Duf 9.8v REx | cagtcg/aattctttggcctaagaaccacctg |
| -9752 | -9774 | Duf 9.8 REx | tgcgataag/aattccaaagttggggtcccatatcgg |
| -9775 | -9750 | Duf 9.8 FEx | gctgcatggcg/aattcccgatatgggaccccaactttggc |
| -14137 | -14161 | Duf 14 RBx | cgtgtttggg/atcccgtttttaagagcagggagcgg |
| -14892 | -14870 | Duf 15 FEx | ggagtgagg/aattcgccggaagcccatcacttagg |
| -15982 | -15959 | Duf 16 FBx | cacgtcgag/gatccaactaagcatcgatttggaatc |
| -15989 | -15970 | Duf 16v FBx | cagtcg/gatcccgaaagcccaactaagcatc |
